# Inhibition of lysosomal degradation increases expression of mutant ADA2 in DADA2 monocytes

**DOI:** 10.1016/j.jaci.2025.06.009

**Published:** 2025-10

**Authors:** Lisa Ehlers, Marjon Wouters, Bethany Pillay, Selket Delafontaine, Mariia Dzhus, Marco Baggio, Tim Niehues, Gregor Dückers, Lieve Sevenants, Kristina Casteels, Lien De Somer, Rik Schrijvers, Steven Vanderschueren, Maarten Jacquemyn, Dirk Daelemans, Anneleen Hombrouck, Eugene P. Chambers, Thomas Tousseyn, Giorgia Bucciol, Patrizia Agostinis, Leen Moens, Isabelle Meyts

**Affiliations:** a Department of Microbiology, Immunology and Transplantation, Laboratory for Inborn Errors of Immunity, KU Leuven, Leuven, Belgium; f Department of Pediatrics, University Hospitals Leuven, KU Leuven, Leuven, Belgium; i Laboratory of Immunobiology, Department of Microbiology and Immunology, KU Leuven, Leuven, Belgium; j Department of Microbiology, Immunology and Transplantation, Allergy and Clinical immunology Research Group, KU Leuven, Leuven, Belgium; b Department of Pediatric Respiratory Medicine, Immunology and Critical Care Medicine, Charité – Universitätsmedizin Berlin, corporate member of Freie Universität Berlin and Humboldt-Universität zu Berlin, Berlin, Germany; c Berlin Institute of Health at Charité – Universitätsmedizin Berlin, Berlin, Germany; d German Center for Child and Adolescent Health, partner site Berlin, Berlin, Germany; e Deutsches Rheuma-Forschungszentrum, an Institute of the Leibniz Association, Berlin, Germany; g Laboratory of Computational and Developmental Biology, Berlin Institute for Medical Systems Biology, Max-Delbrück-Centrum for Molecular Medicine in the Helmholtz Association, Berlin, Germany; h Helios Children's Hospital, Krefeld, Germany; k Department of General Internal Medicine, Research Department Microbiology, Immunology, and Transplantation, Laboratory of Clinical Infectious and Inflammatory Disorders, University Hospitals Leuven, Leuven, Belgium; o Department of Pathology, University Hospitals Leuven, Leuven, Belgium; l KU Leuven Department of Microbiology, Immunology and Transplantation, Molecular Genetics and Therapeutics in Virology and Oncology Research Group, Rega Institute for Medical Research, Leuven, Belgium; m Vanderbilt University Medical Center, Nashville, Tenn; n DADA2 Foundation, Nashville, Tenn; p Cell Death Research and Therapy Lab, Department of Cellular and Molecular Medicine, Center for Cancer Biology, VIB-KU Leuven, Leuven, Belgium

**Keywords:** Deficiency of ADA2, cytopenia, hydroxychloroquine, lysosomal degradation, TLR9 signaling

## Abstract

**Background:**

Deficiency of adenosine deaminase 2 (DADA2) is an inborn error of immunity causing vasculitis and bone marrow failure. Bone marrow failure is mostly unresponsive to TNF-α inhibitors. The limited understanding of the pathomechanisms driving the disease impedes the development of new treatment options. Unlike cellular model systems expressing pathogenic *ADA2* variants, primary monocytes from patients with DADA2 lack ADA2 protein expression.

**Objectives:**

This study aimed to analyze the role of protein degradation in the pathogenesis of DADA2 and the therapeutic potential of the lysosomotropic drug hydroxychloroquine in the treatment of patients with DADA2.

**Methods:**

ADA2 protein expression in CD14^+^ monocytes from healthy controls (n = 8) and patients with DADA2 (n = 11) was determined by Western blot after inhibition of lysosomal and proteasomal degradation, as well as after hydroxychloroquine treatment *in vivo* in 1 patient with DADA2. Lipidation of microtubule associated protein 1 light chain 3 beta (LC3B) was analyzed as a measure of autophagic activity. Clinical and laboratory data were recorded in cytopenic patients with DADA2 treated with hydroxychloroquine, 200 mg per day.

**Results:**

We demonstrated that inhibition of lysosomal degradation restores ADA2 protein expression in DADA2 monocytes *in vitro*. DADA2 monocytes exhibited increased autophagic activity. We observed clinical improvement in 2 cytopenic patients with DADA2 who were treated with hydroxychloroquine, and we showed a concomitant increase in ADA2 protein levels in monocytes from one of these patients *in vivo*.

**Conclusion:**

We identified lysosomal protein degradation of ADA2 as a pathomechanism of DADA2 and introduced hydroxychloroquine as a potential treatment option in patients with DADA2 with refractory cytopenia.

## Graphical abstract

**Figure:**
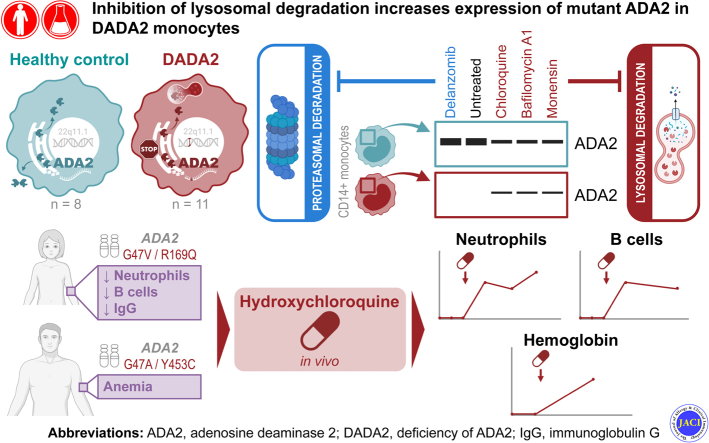

## Introduction

Deficiency of ADA2 (DADA2) is a rare inborn error of immunity caused by biallelic mutations in the *ADA2* gene.^1^ The disease manifests with a diverse phenotype of vasculitis, immunodeficiency, and bone marrow failure.^2^ TNF-α inhibitors (TNFis) alleviate vasculitic symptoms, but patients with bone marrow failure mostly require hematopoietic stem cell transplantation, which is complicated by a high incidence of graft failure.^3^ The pathophysiology of DADA2 is poorly understood and has mostly been studied in models requiring overexpression of mutant ADA2. In primary DADA2 monocytes expressing *ADA2* missense variants, however, ADA2 protein expression is minimal to absent despite normal mRNA expression,^4^, ^5^, ^6^ suggesting rapid degradation of mutant ADA2 protein. Here, we show for what we believe to be the first time that inhibition of lysosomal degradation increases protein expression of endogenous mutant ADA2 in monocytes from patients with DADA2. In addition, we report 2 patients with DADA2 whose cytopenia improved after initiation of treatment with the lysosomotropic agent hydroxychloroquine.

## Results and discussion

**Fig 1:**
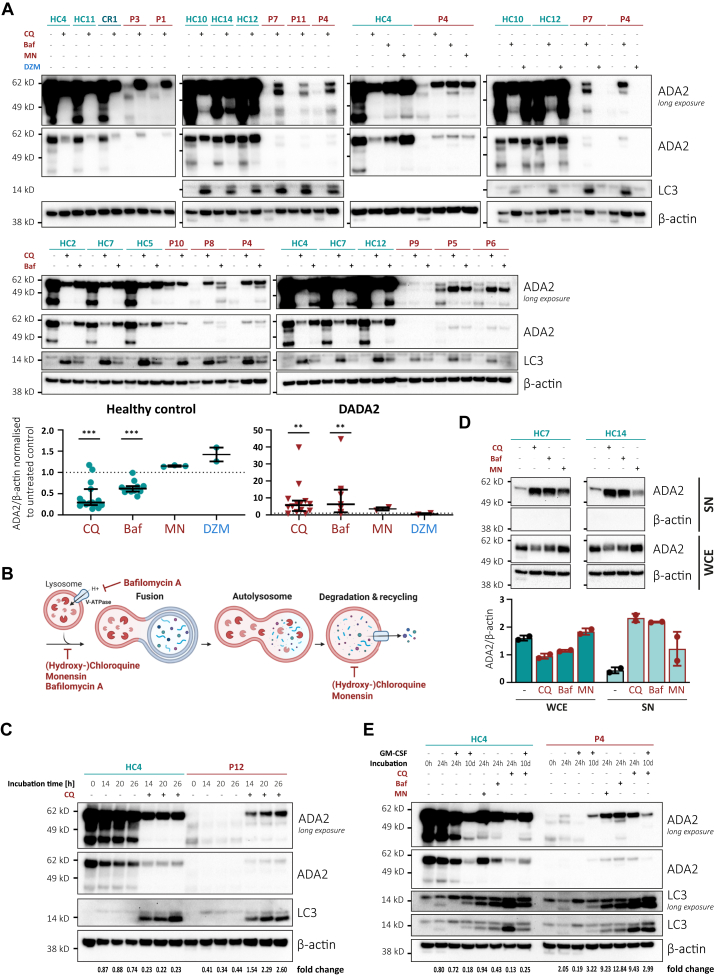
**A, C-E,** ADA2 protein expression by Western blot in whole cell extracts (WCEs) or supernatants (SNs) of CD14^+^ monocytes or PBMCs (**D**) from HCs, DADA2 heterozygous carriers (CRs) or patients with DADA2 (P) after 20 to 24 hours of incubation unless indicated. Dot plots show medians and interquartile ranges. **E,** CD14^+^ monocytes were incubated with or without GM-CSF and inhibitors of lysosomal degradation, as labeled for the indicated culture periods. Fold changes refer to ADA2 normalized to β-actin versus at hour zero. **B,** Mechanism of action of different inhibitors of lysosomal degradation. ∗∗*P* < .01, ∗∗∗*P* < .001. *Baf,* Bafilomycin A; *CQ*, chloroquine; *DZM*, delanzomib; *MN*, monensin.

To test whether mutant ADA2 undergoes autophagic degradation, we treated CD14^+^ monocytes from healthy controls (HCs), heterozygous carriers, and patients with DADA2 with different inhibitors of lysosomal degradation. We observed a strong increase in ADA2 protein expression in the patients’ cells (Fig 1, *A-C*), including the cells of patient P5 and patient P6, who harbored the c.del1240-1442 variant resulting in deletion of exon 9 (Table I). Incubation with the proteasome inhibitor delanzomib did not alter mutant ADA2 expression (Fig 1, *A*). In HC monocytes, we observed a reduction in intracellular ADA2 levels after initiation of treatment with chloroquine and bafilomycin A that was due to an increase in protein secretion (Fig 1, *D*). Macrophage differentiation by GM-CSF also restored ADA2 expression in ADA2-deficient cells (Fig 1, *E*).

**Table I:** Cohort characteristics

Patient ID	Age (y)	Sex	Genotype	Predominant phenotype
P1	9	F	c.140G>T/c.506G>A	Bone marrow failure, hypogammaglobulinemia, hepatosplenomegaly
			p.G47V/p.R169Q	
P2	15	M	c.140G>C/c.1358A>G	Intraventricular hemorrhage, anemia, livedo
			p.G47A/p.Y453C	
P3	6	F	c.140G>T/c.506G>A	Hypogammaglobulinemia, hepatosplenomegaly, stroke
			p.G47V/p.R169Q	
P4	26	M	c.140G>T/c.140G>T	Hypogammaglobulinemia, hepatosplenomegaly
			p.G47V/p.G47V	
P5	12	M	c.973-2A>G/c.del1240-1442	Vasculitis, hepatosplenomegaly, hypogammaglobulinemia
			Splice site (intron 6)/del exon 9	
P6	14	F	c.973-2A>G/c.del1240-1442	Vasculitis, warts, hypogammaglobulinemia, arthralgia
			Splice site (intron 6)/del exon 9	
P7	32	F	c.973-2A>G/c.506G>A	Stroke, hypertension
			Splice site (intron 6)/p.R169Q	
P8	16	M	c.139G>A/c.139G>A	Stroke, vasculitis
			p.G47R/p.G47R	
P9	10	F	c.973-2A>G/c.973-2A>G	Vasculitis, hypogammaglobulinemia
			Splice site (intron 6)/ splice site (intron 6)	
P10	50	F	c.973-2A>G/c.506G>A	Vasculitis, leukopenia
			Splice site (intron 6)/p.R169Q	
P11	26	M	c.140G>T/c.del1240-1442	Bone marrow failure, hypogammaglobulinemia, hepatosplenomegaly
			p.G47V/del exon 9	
P12	22	M	c.506G>A/c.506G>A	Neutropenia, anemia, liver disease
			p.R169Q/p.R169Q	
CR1	38	F	WT/c.140G>T	
			WT/p.G47V	
CR2	42	M	WT/c.506G>A	
			WT/p.R169Q	

This cohort of patients with DADA2 has also been presented in the context of another study conducted by this research group.^6^ The patient IDs have been reassigned in this report to improve readability.

*F*, Female; *ID*, identifier; *M*, male; *WT*, wild type.

Given the pronounced effects of inhibition of lysosomal degradation *in vitro*, we hypothesized that the lysosomotropic agent hydroxychloroquine might be beneficial in the treatment of patients with DADA2.

**Fig 2:**
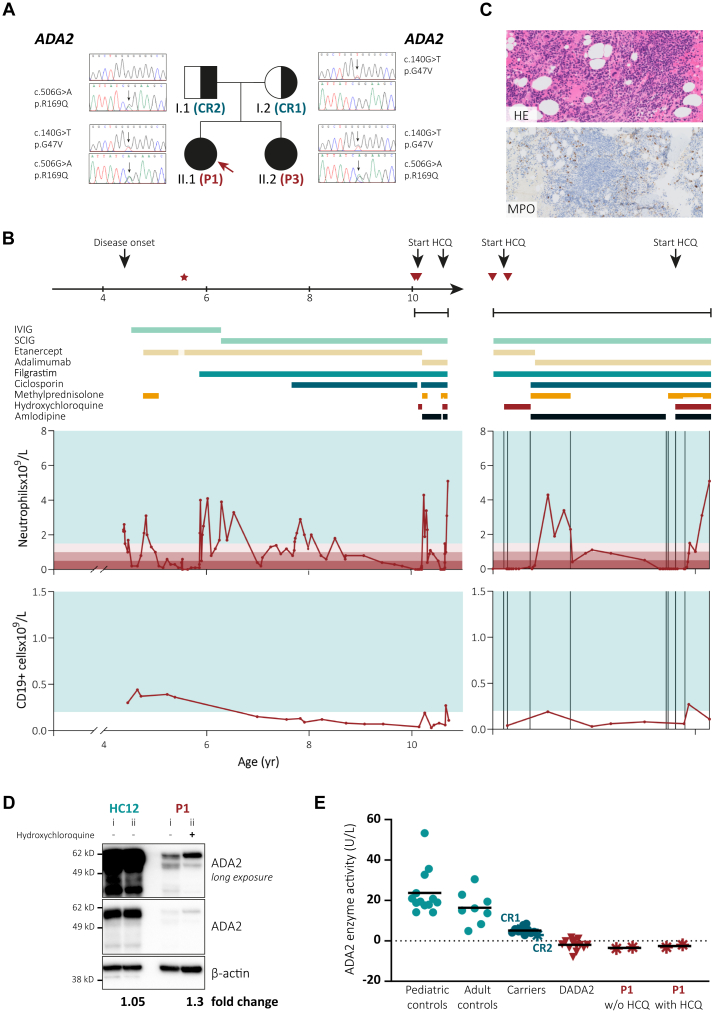
Case report of patient P1. **A,** Pedigree and genotype. **B,** Clinical data over time. Star marks bone marrow biopsy shown (**C**). Triangles indicate blood draws before (*i*) and during (*ii*) hydroxychloroquine (HCQ) treatment shown (**D**). Green band indicates the age-specific reference range. **C,** Bone marrow biopsy: hematoxylin and eosin (HE) stain and immunohistochemistry for myeloperoxidase (MPO). **D**, Monocytic ADA2 protein expression by Western blot in patient with DADA2 designated as patient P1 before (*i*) and 4 days after initiation of treatment with HCQ (*ii*). Fold change represents ADA2 expression normalized to β-actin from time point (*ii*) to time point (*i*). (E,) Serum ADA2 enzyme activity. *CR*, Carrier; *IVIG*, intravenous immunoglobulin; *SCIG*, subcutaneous immunoglobulin.

Patient P1 of our cohort was diagnosed with DADA2 at age 4 years, when she presented with recurrent fevers, livedoid rash, arthralgia, hypogammaglobulinemia, and recurrent infections (Fig 2, *A*). At age 5 years, she developed neutropenia (Fig 2, *B*). A bone marrow biopsy showed severe reduction of the myeloid lineage and absent maturation of polymorphonuclear neutrophils (Fig 2, *C*). The patient began taking filgrastim, and her neutrophil counts temporarily improved.

At the time of this report, at age 10 years, the patient’s neutropenia had relapsed and she developed B lymphopenia with absence of class-switched CD27^+^IgM^–^IgD^–^ memory B cells. She was receiving etanercept (30 mg per week), ciclosporin (90 mg per day), filgrastim (200 μg per day [ie, 7 μg/kg per day]), and subcutaneous immunoglobulin (3 g per week). At this point, her ciclosporin treatment was paused, and guided by the effects of chloroquine treatment on DADA2 monocytes observed *in vitro*, we started giving her hydroxychloroquine (200 mg per day) as an alternative immunomodulatory agent (Fig 2, *B*). We isolated CD14^+^ monocytes from the patient’s peripheral blood before treatment initiation and 4 days after the hydroxychloroquine had been started and analyzed ADA2 protein expression by Western blot. We observed a 1.3-fold increase in intracellular ADA2 protein levels in the patient’s monocytes but not in the monocytes of an untreated HC that were isolated at both time points in parallel (Fig 2, *D*). The patient’s serum ADA2 enzyme activity did not change after initiation of hydroxychloroquine treatment (Fig 2, *E*). Four weeks later, the hydroxychloroquine was discontinued on account of neutropenic fever. Her TNFi treatment was switched from etanercept to adalimumab. Shortly after, both her neutrophil and B-cell counts recovered. In the following months, we observed another decline in her neutrophil and B-cell counts. Because of the positive evolution of her episodes of cytopenia after the first course of hydroxychloroquine, we restarted the treatment. Twelve days after the patient began taking hydroxychloroquine, her neutrophil and B-cell counts started to rise to normal values (Fig 2, *B*). The patient tolerated the treatment well and her clinical state improved while methylprednisolone was being tapered to 4 mg per day. Filgrastim was discontinued 1 week before the last follow-up, and her neutrophil counts remained normal.

Meanwhile, we learned of the case of a yet unreported German patient (patient P2) who presented with anemia and low-normal white blood cell and platelet counts in 2015. After initiation of treatment with hydroxychloroquine, 200 mg per day, his cell counts normalized (Table II). Although the patient was stable during this monotherapy, etanercept (50 mg per week) was later added to his treatment because of the increasing evidence that TNFis prevent strokes in patients with DADA2.

**Table II:** Clinical evolution of patient P2 after initiation of hydroxychloroquine treatment

Laboratory value	Baseline	After 3 mo
Hemoglobin level (g/dL)	7.4	12.1
Red blood cell count (cells × 10^12^/L)	3.7	5.5
White blood cell count (cells × 10^9^/L)	4.7	7.1
Platelet count (cells × 10^9^/L)	186	319
Neutrophil count (cells ×10^9^/L)	2.9	4.1
C-reactive protein level (mg/L)	29	<3

Laboratory values shown are those of patient P2 before initiation of hydroxychloroquine treatment (baseline) and after 3 months of hydroxychloroquine treatment.

The case report of patient P1 highlights the limited treatment options for patients with DADA2 who present with bone marrow failure.^3^^,^^7^ The recent description of a potential role of ADA2 as a lysosomal protein directed our attention to the intracellular processing of endogenous mutant ADA2.^5^^,^^8^^,^^9^ Our data indicate that mutant ADA2 expressed in DADA2 monocytes undergoes rapid degradation in the lysosomes. This finding introduces a new pathomechanism besides impaired secretion and enzymatic activity of ADA2, an aspect that is not reflected in HEK293T cells transfected with pathogenic *ADA2* variants.^4^

Hydroxychloroquine inhibits lysosomal degradation and is widely used in the treatment of inflammatory diseases.^10^ Patient P1 tolerated the treatment well, but her neutropenia did not immediately improve. Given the drug’s half-life of 40 days, we suspected the subsequent improvement to be within the context of the hydroxychloroquine therapy. Hydroxychloroquine is known for its slow onset of immunomodulatory action, and it has been hypothesized that intracellular accumulation of the drug is required for its non-antimalarial effects.^10^^,^^11^ As the patient received multiple treatments in rapid succession, it was difficult to link the resolution of her neutropenia to a single reagent. Glucocorticoids are well known for causing neutrophilic leukocytosis, but a beneficial effect on B-cell counts is unlikely.^12^ We restarted the treatment with hydroxychloroquine when her neutropenia relapsed several months later and remained unresponsive to filgrastim. Again, we observed an increase in her neutrophil and B-cell counts with some delay after initiation of the hydroxychloroquine treatment. Before the hydroxychloroquine treatment, the patient had already been receiving methylprednisolone for a colitis flare without any effect on her cell counts. At the last follow-up, her neutrophil counts were normal despite continuous taper of the glucocorticoids. The case report of patient P2 strongly supports the idea of a beneficial effect of hydroxychloroquine in cytopenic patients with DADA2. Because the patient’s cell counts improved when receiving monotherapy with hydroxychloroquine, we can exclude the contribution of other medications in his case.

The limited number of patients and the polypharmacy in 1 of the 2 cases are limitations of this study. A thorough assessment of the efficacy of the drug in improving the complex phenotype of patients with DADA2 would also require a control group. Given the diverse manifestations and the rarity of the disease, a multicenter study will be needed to carefully address these aspects.

**Fig 3:**
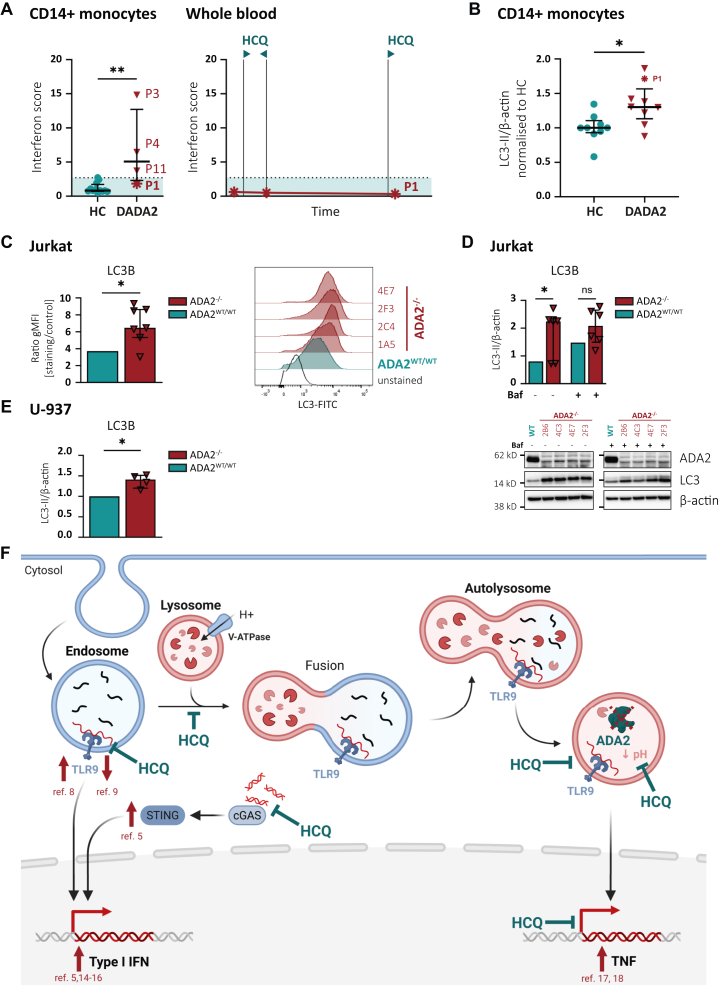
**A,** Interferon (IFN) score by quantitative PCR in CD14^+^ monocytes from HCs and patients with DADA2 (all without hydroxychloroquine [HCQ] treatment) as well as in the whole blood of patient P1 before and with HCQ therapy. **B,** LC3-II expression after 24 hours of incubation without treatment determined by Western blot normalized to β-actin in CD14^+^ monocytes. **C,** Autophagic LC3 level measured by flow cytometry. **D** and **E,** Normalized expression of LC3-II by Western blot. Dot plots show medians and interquartile ranges. **F,** Possible mechanisms of action of HCQ in DADA2. Red arrows indicate proposed pathomechanisms of DADA2. Absence of ADA2 can lead to enhanced sensing of CpG DNA by TLR9^8^ or reduced TLR9 activation owing to lack of deoxyadenosine deamination.^9^ DADA2 is characterized by an increased type I IFN and TNF-α response.^5^^,^^14^, ^15^, ^16^, ^17^, ^18^*In vitro*, the elevated type I IFN response was rescued by knockout of STING.^5^ HCQ has been shown to interfere with these pathways at the indicated sites of action. ∗*P* < .05; ∗∗*P* < .01. *Baf*, Bafilomycin; *FITC*, fluorescein isothiocyanate; *gMFI*, geometric mean fluorescence intensity; *ns*, not significant; *WT*, wild type.

This study establishes 2 main principles: (1) the absence of ADA2 protein expression in DADA2 monocytes owing to lysosomal degradation and (2) the clinical benefit of hydroxychloroquine treatment in patients with DADA2 with cytopenia. Although our approach was guided by the assumption that the efficacy of hydroxychloroquine in patients with DADA2 could be attributed to a partial restoration of ADA2 protein expression, we must not neglect the numerous proposed mechanisms of action of the drug. Hydroxychloroquine has been used most widely in the treatment of SLE, a disease characterized (like DADA2) by an elevated type I interferon signature.^13^, ^14^, ^15^, ^16^ To determine whether the beneficial effect of hydroxychloroquine was mediated by an attenuation of interferon signaling, we measured the interferon signature in the blood of patient P1 before and after initiation of hydroxychloroquine treatment (Fig 3, *A*^5^^,^^8^^,^^9^^,^^14^, ^15^, ^16^, ^17^, ^18^). The patient exhibited a normal interferon score at all time points, highlighting the discrepancy between the inflammatory activity and the clinical severity in patients with a predominant phenotype of bone marrow failure. Because autophagy is closely intertwined with the immune response,^19^^,^^20^ we hypothesized that autophagic dysregulation might contribute to DADA2 pathogenesis. By analyzing autophagic flux, we observed increased levels of lipidated LC3B in CD14^+^ monocytes of patients with DADA2 (Fig 3, *B*) at baseline, pointing to an upregulation of autophagy in DADA2. We confirmed this finding in ADA2^–/–^ Jurkat and U-937 cell lines (Fig 3, *C-E*).

Besides modulation of autophagy, chloroquine has been shown to influence several pathways affected in ADA2-deficient cells (Fig 3, *F*). Recently, 2 research groups have proposed that ADA2 mediates the regulation of DNA sensing by Toll-like receptor (TLR) 9.^8^^,^^9^ This process requires acidification of the endosomal-lysosomal compartments and is impaired by chloroquine.^21^ Moreover, chloroquine has also been shown to interfere with binding of CpG DNA to TLR9.^22^

Similarly, hydroxychloroquine affects DNA sensing by cyclic GMP-AMP synthase (cGAS).^23^ Besides type I interferon signaling, chloroquine has also been shown to reduce gene expression of TNF-α, a key cytokine in the pathogenesis of DADA2.^17^^,^^18^^,^^24^ Thus, the restoration of ADA2 protein expression and attenuation of the clinical phenotype in DADA2 may represent independent effects of the hydroxychloroquine.

Nevertheless, our case reports highlight the potential of drug repurposing to expand the treatment options for patients with DADA2 with cytopenia unresponsive to TNFis.

In conclusion, we have demonstrated that inhibition of lysosomal degradation increased protein expression of mutant ADA2 in CD14^+^ monocytes from patients with DADA2 *in vitro* and in a DADA2 patient with treatment-refractory neutropenia *in vivo*. We have reported the clinical improvement of 2 patients with DADA2 treated with the lysosomotropic agent hydroxychloroquine. Our data provide a strong basis to pursue further research into the therapeutic implications of altered lysosomal protein degradation in DADA2.

**Key messages**

• Protein expression of mutant ADA2 in monocytes from patients with DADA2 is restored by inhibition of lysosomal protein degradation.
• Hydroxychloroquine treatment improved the clinical phenotype of 2 patients with DADA2 presenting with cytopenia.

## Disclosure statement

Supported by the 10.13039/501100000781European Research Council under the European Union’s Horizon 2020 research and innovation program (grant 948959) (MORE2ADA2). I.M. is a senior clinical investigator at the Research Foundation–Flanders and is supported by KU Leuven (C1 grant C16/18/0070), Research Foundation–Flanders (grant G0B5120N), and the Jeffrey Modell Foundation. This work was supported by ERN-RITA. L.E. was supported by a PhD fellowship from the Research Foundation–Flanders (grant 11E0123N) and is a fellow of the BIH Charité Junior Clinician Scientist Program funded by the Charité–Universitätsmedizin Berlin, and the Berlin Institute of Health at Charité. S.D. was supported by a PhD fellowship from the Research Foundation–Flanders (grant 11F4421N). R.S. is a Research Foundation–Flanders senior clinical investigator fellow (grant 1805523N). P.A. is supported by grants from the Flemish Research Foundation (grant G0A3320N), KU Leuven (grant C14/21/095) InterAction consortium, the EOS MetaNiche consortium (grant 40007532), the iBOF/21/053 ATLANTIS network, and the EOS DECODE consortium (grant 30837538). The sponsors were not involved in the study design collection, analysis, and interpretation of the data, or the writing of this report.

Disclosure of potential conflict of interest: L. Ehlers and I. Meyts have a patent application related to this work (patent no. PCT/EP2024/078038). The rest of the authors declare that they have no relevant conflicts of interest.
