# Supplementary material for: Inhibition of lysosomal degradation increases expression of mutant ADA2 in DADA2 monocytes
Source: J Allergy Clin Immunol. 2025 Oct;156(4):1111–9. doi: 10.1016/j.jaci.2025.06.009 (PMC12499373; doi:10.1016/j.jaci.2025.06.009)
Supplement: Supplementary Data [file mmc1.docx]

Online Repository

# Methods

## Study details

All patients or their parents provided written informed consent. The patients were treated at University Hospitals Leuven and their parents were included as heterozygous carriers. The second reported case describes a patient that was treated at Helios Children's Hospital, Krefeld, Germany. Healthy donors were recruited at University Hospitals Leuven and KU Leuven. This study was performed in accordance with the ethical standards as laid down in the 1964 Declaration of Helsinki and was approved by the Ethics Committee for Research of Leuven University Hospitals (project number S63077, S63807).

## Sanger sequencing

Genomic DNA samples were prepared from heparinised peripheral blood following the instructions of the QIAamp DNA Blood Mini kit (#51104; QIAGEN). Primers were designed with the help of Oligo Primer Analysis Software version 7 (Molecular Biology Insights). ADA2-specific gDNA amplification was performed using Platinum™ SuperFi™ PCR Master Mix (#12358010; Thermo Fisher Scientific). PCR products were purified using the QIAquick PCR purification kit (#28106; QIAGEN). Sanger sequencing was performed on an ABI 3730 XL Genetic Analyzer (Applied Biosystems) at LGC Genomics (Berlin, Germany). Sequencing data were analysed using Chromas 2.6.5 (http://www.technelysium.com.au).

## Cell culture

Peripheral blood mononuclear cells (PBMCs) were isolated by density gradient centrifugation using Lymphoprep™ (#1114546; PROGEN) in SepMate™ isolation tubes (#85450; STEMCELL Technologies) according to the manufacturer’s instructions. Prior to monocyte isolation, the final centrifugation step was performed at 300xg for 10 minutes at 4°C. CD14+ monocytes were isolated magnetically by positive selection using human CD14 MicroBeads (#130-050-201; Miltenyi Biotec) on LS columns (#130-042-401; Miltenyi Biotec) according to the manufacturer’s protocol. The cells were eluted into complete medium (RPMI 1640 medium (#61870044; Gibco) supplemented with 10% fetal calf serum (FCS) (#S181BH-500, Biowest) and 1% penicillin-streptomycin (#15140122; Gibco). Purity > 97% of CD14+ monocytes after magnetic sorting was verified by flow cytometry (anti-CD14-FITC clone MφP9, 1:40, #345784; BD Biosciences). For macrophage differentiation, 3x10^5^ CD14+ monocytes were seeded in a 12-well plate in 1 mL complete medium containing 20 ng/mL GM-CSF (#300-03; Peprotech). The cells were differentiated for ten days, with medium changes every three days. U-937 and Jurkat cells (purchased from ATCC) were cultured in complete RPMI medium.

## Generation of ADA2^-/-^ cell lines by CRISPR/Cas9

Single-guide RNAs targeting ADA2 from the human CRISPR Brunello library (#73179; addgene) were cloned into the lentiCRISPRv2 puro plasmid.^E1^ lentiCRISPRv2 puro was a gift from Brett Stringer (plasmid #98290; addgene; http://n2t.net/addgene:98290; RRID: Addgene_98290). U-937 and Jurkat cells were transfected by electroporation using the Neon™ Transfection System (#MPK5000; Thermo Fisher Scientific) according to the manufacturer’s instructions: 2x10^5^ cells were resuspended in Resuspension Buffer R and mixed with 1 µg plasmid DNA. Electroporation was achieved with the following conditions: 1325V, 10ms, 3 pulses. After electroporation, the cells were directly transferred into 500 µL prewarmed complete RPMI 1640 medium. On day 1 after transfection, puromycin (#ant-pr-1; InvivoGen) was added at a final concentration of 1 µg/mL. After 36h, cells were seeded at 1 cell/well in 100 µL RPMI 1640 medium supplemented with 20% FCS in a round bottom 96-well plate. After 14 days, the clones were screened for knock out of ADA2 by western blot.

## Inhibition of lysosomal degradation

To inhibit autophagy, cells were treated with 50 µM chloroquine (#C6628; Sigma-Aldrich), 100 nM bafilomycin A1 (#B1793; Sigma-Aldrich) or 2 µg/mL monensin (GolgiStop™, #554724, BD Biosciences) for 24 hours. Proteasome inhibition was achieved by 24-hour incubation with 200 nM delanzomib (#S1157; Selleck Chemicals GmbH). ADA2 expression in the whole cell lysates or cell supernatant was determined by western blot.

## Immunoblotting

Adherent cells were detached with trypsin-EDTA (0.05%) (#25300062; Gibco) for 5 minutes at 37°C and the reaction was blocked by addition of complete medium. Cells were pelleted by centrifugation at 400xg for 5 minutes at 4°C. Whole cell lysates were obtained by lysing 1x10^6^ cells in 25 µL RIPA buffer (150 mM NaCl, 1% Triton X-100, 0.5% sodium deoxycholate, 0.1% SDS, pH 8.0) or NP-40 lysis buffer (150 mM NaCl, 50 mM Tris-HCl, 1% NP-40, pH 7.4) containing protease inhibitor (#78429; Thermo Fisher Scientific) for 30 minutes on ice, followed by centrifugation at 13,500xg for 20 minutes at 4°C. Bolt™ LDS sample buffer (#B0007; Thermo Fisher Scientific) mixed with Bolt™ Sample Reducing Agent (#B0009; Thermo Fisher Scientific) was added to the samples prior to gel electrophoresis. 10 µg protein per sample were put on the gel. The western blots shown in **Figure 2D** were done with 20 µg protein per sample. Proteins were transferred onto PVDF transfer membranes. The membrane was probed with the following primary antibodies: anti-ADA2 (clone EPR25430-131, #ab288296, 1:1000; abcam), anti-LC3B (#ab51520, 1:1000; abcam; RRID: AB_881429), anti-β-actin (clone: AC-15, #A5441, 1:9,000; Sigma-Aldrich; RRID: AB_476744) at 4°C overnight or at room temperature for two hours. The membranes were washed and incubated with the respective HRP-coupled secondary antibodies for one hour at room temperature: goat anti-rabbit IgG H&L (#ab205718, 1:5000; abcam; RRID: AB_2819160) or Goat Anti-Mouse IgG (H + L) (#71045, 1:5000; Sigma-Aldrich; RRID: AB_11211441). Protein expression was visualized by enzymatic chemiluminescence using Pierce^TM^ ECL western blotting substrate (#32106; Thermo Fisher Scientific) or SuperSignal™ West Pico PLUS Chemiluminescent Substrate (#34580; Thermo Fisher Scientific) in a ChemiDoc XRS+ Imaging System (Bio-Rad).

## ADA2 enzyme activity

Adenosine deaminase 2 enzyme activity was determined human serum. Deaminase activity was measured in a colorimetric assay adapted from Giusti and Galanti.^E2^ Erythro-9-(2-hydroxy-3-nonyl) adenine (EHNA) (#E114; Sigma-Aldrich) was used to inhibit ADA1 activity. Triplicate measurements were performed for all samples.

## qPCR

For analysis of whole blood samples, total RNA was extracted from PAXgene RNA tubes using the PAXgene Blood RNA Kit, v2 (PreAnalytiX, Qiagen/ BD). Alternatively, 5-10x10^5^ CD14+ monocytes were lysed in TRIzol™ Reagent (#15596018; Thermo Fisher Scientific) for 3 minutes at room temperature and homogenized before storage at -80°C. RNA was extracted using the PureLink™ RNA Mini Kit (#12183018A; Thermo Fisher Scientific) according to the manufacturer’s instruction. cDNA was generated from 20 ng RNA using the SuperScript™ VILO™ cDNA Synthesis Kit (#11754050; Thermo Fisher Scientific). Quantitative polymerase chain reaction (qPCR) analysis was performed with SsoAdvanced™ Universal SYBR® Green Supermix (#1725271; Bio-Rad Laboratories) and the following primers:

IFI27_F_TCGCCTCGTCCTCCATAGCAG;

IFI27_R_ AGTAGAACCTCGCAATGACAGCC;

IFI44L_F_ATCTTAAAAGGTTGTATGCCAGA;

IFI44L_R_ACTTGCTTCACTTTTGCCAA; IFIT1_F_ATGAGTACAAATGGTGATGA;

IFIT1_R_AATTCAATCTGATCCAAGAC; ISG15_F_GGTGGACAAATGCGACGAACCTC;

ISG15_R_CACACCCTCCAGCCCGCTCA; RSAD2_F_ GCGTCAACTATCACTTCACTCG; RSAD2_R_ CAGGTATTCTCCCCGGTCT; SIGLEC1_F_ TCTTGCCCAAGCTTCTCCTC; SIGLEC1_R_GTAGTACCAGATGGCCGTGA; GAPDH_F_GTCTCCTCTGACTTCAACAGCG; GAPDH_R_ACCACCCTGTTGCTGTAGCCAA. For all conditions, three technical replicates were measured. The experiment was run on a QuantStudio™ 3 Real-Time PCR System (Thermo Fisher Scientific) and analyzed using the QuantStudio™ Design & Analysis Software v1.5.2. The relative abundance of the respective genes was normalized to the expression level of *GAPDH*. and different conditions were compared using the 2^-ΔΔCt^ method.^E3^ The median fold change of the six interferon-stimulated genes *IFI27*, *IFI44L*, *IFIT1*, *ISG15*, *RSAD2*, *SIGLEC1* when compared to expression in healthy control samples was used to create an interferon score for each individual.^E4^

## Flow cytometry

Jurkat cells were placed on ice at the end of the incubation period and washed with PBS prior to staining. Autophagic LC3 was detected using the Guava® Autophagy LC3 Antibody-Based Detection Kit (#SKU FCCH100171; Cytek Biosciences) according to the manufacturer’s instructions. The measurements were performed on a Symphony flow cytometer (BD Biosciences). The data were analyzed with FlowJo software (version 10.8.1). For analysis, debris was excluded by forward and sideward scatter and singlets were identified before further analysis.

## Statistical analysis

ADA2 and LC3-II protein levels were normalized to β-actin. To determine the effect of inhibition of lysosomal degradation on ADA2 expression, samples were compared to paired untreated control samples. For analysis for LC3-II expression, DADA2 patient samples were normalized to mean healthy control values from the same blot to allow for inter-blot comparability. Statistical analyses were performed with R and GraphPad Prism. Shapiro-Wilk normality test was performed to test for normality of the samples. Where a normal distribution could be assumed, samples were compared using unpaired t test and one sample t test, respectively. For ADA2 expression, the nonparametric Wilcoxon matched-pairs signed rank test was used.

## Data Sharing Statement

We provide a separate file showing the unedited full blots from all displayed experiments as data supplement available with the online version of this article.

# References

1. Doench JG, Fusi N, Sullender M, Hegde M, Vaimberg EW, Donovan KF, et al. Optimized sgRNA design to maximize activity and minimize off-target effects of CRISPR-Cas9. Nat Biotechnol. 2016;34:184–91.
2. Illingworth J. Methods of enzymatic analysis: Third edition: Editor-in-Chief: Hans Ulrich Bergmeyer. Verlag Chemie, 1983 (vols I–III), 1984 (vols IV & V) DM258 each volume or DM2240 vols I–X inclusive. Biochemical Education. 1985;13:38–38.
3. Livak KJ, Schmittgen TD. Analysis of relative gene expression data using real-time quantitative PCR and the 2(-Delta Delta C(T)) Method. Methods. 2001;25:402–8.
4. Rice GI, Melki I, Frémond ML, Briggs TA, Rodero MP, Kitabayashi N, et al. Assessment of Type I Interferon Signaling in Pediatric Inflammatory Disease. J Clin Immunol. 2017;37:123–32.
