# Supplementary material for: Inhibition of lysosomal degradation increases expression of mutant ADA2 in DADA2 monocytes
Source: J Allergy Clin Immunol. 2025 Oct;156(4):1111–9. doi: 10.1016/j.jaci.2025.06.009 (PMC12499373; doi:10.1016/j.jaci.2025.06.009)

Figure 1A

anti-ADA2 (ab288296) + goat anti-rabbit (ab205718)

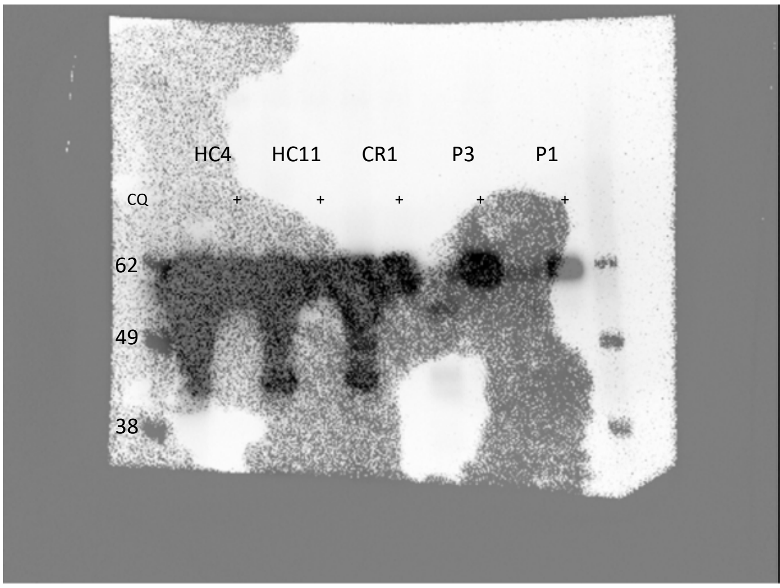

anti-β-actin (AC-15) + goat anti-mouse (#71045)

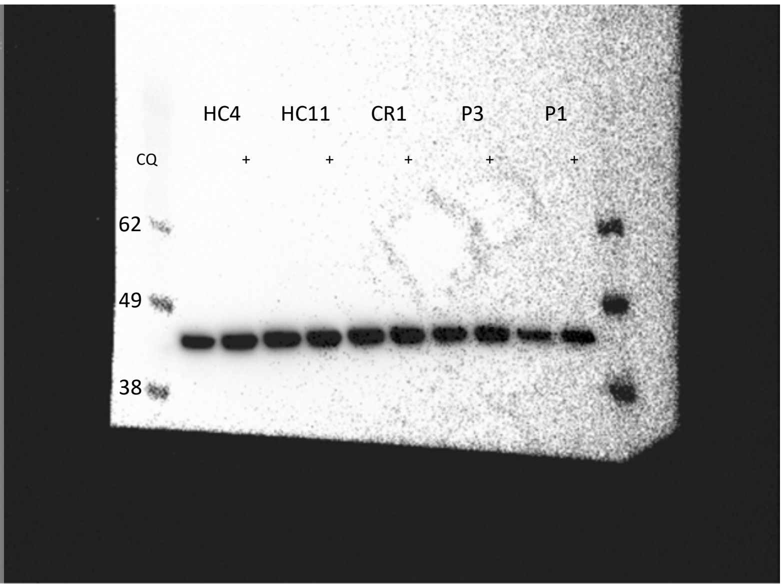

anti-ADA2 (ab288296) + goat anti-rabbit (ab205718)

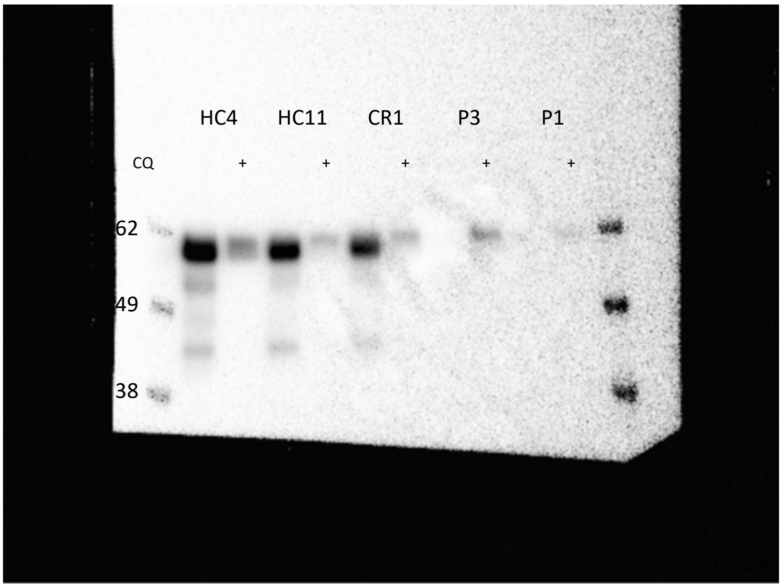

anti-ADA2 (ab288296) + goat anti-rabbit (ab205718)

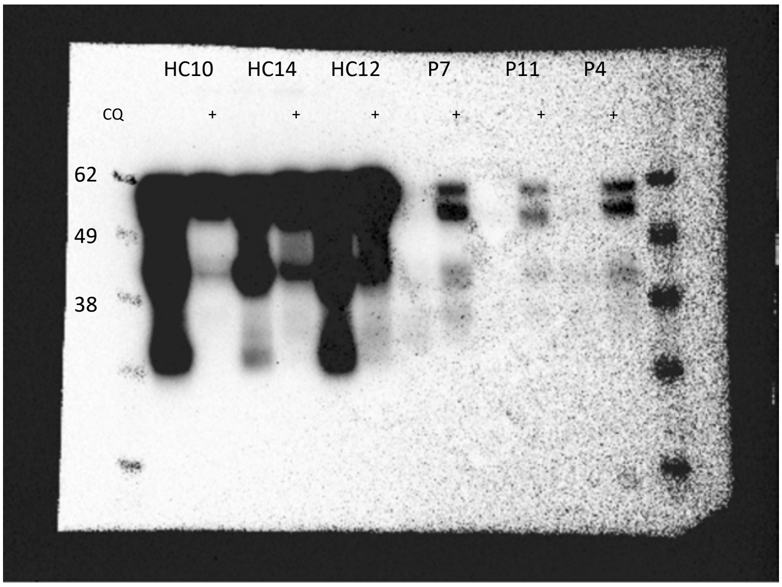

anti-β-actin (AC-15) + goat anti-mouse (#71045)

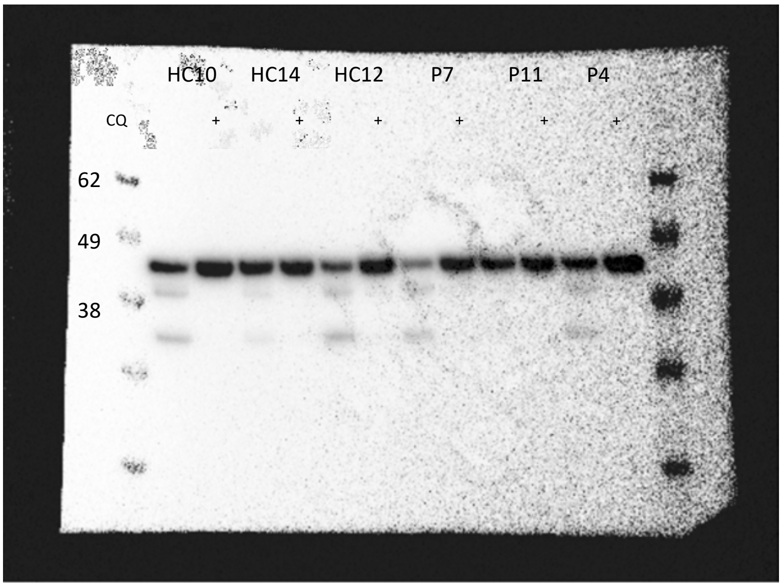

anti-ADA2 (ab288296) + goat anti-rabbit (ab205718)

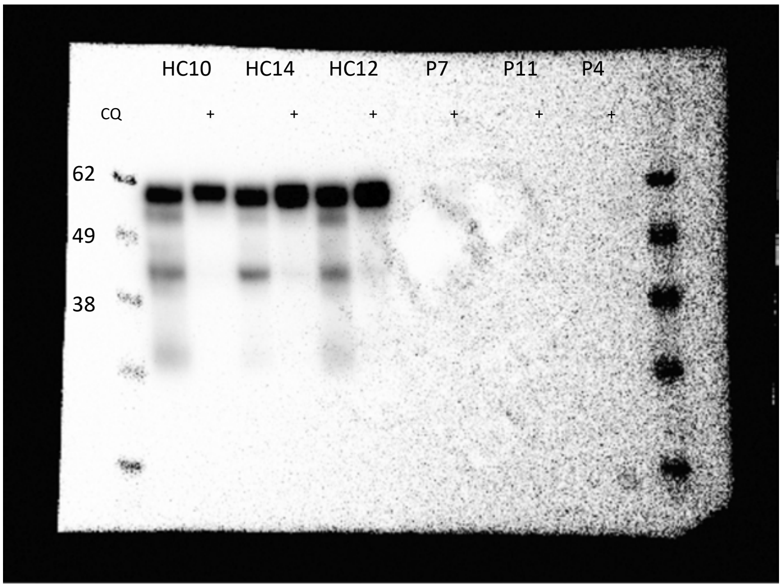

anti-LC3B [ab51520] + goat anti-rabbit (ab205718)

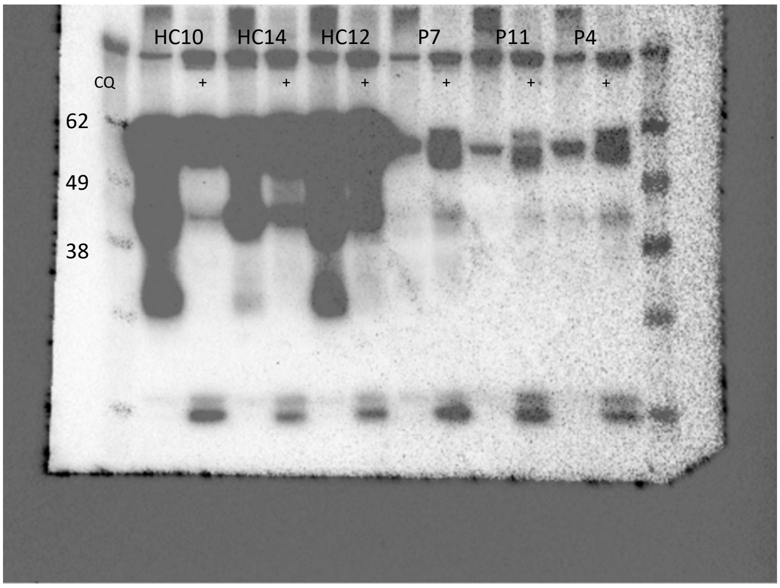

anti-ADA2 (ab288296) + goat anti-rabbit (ab205718)

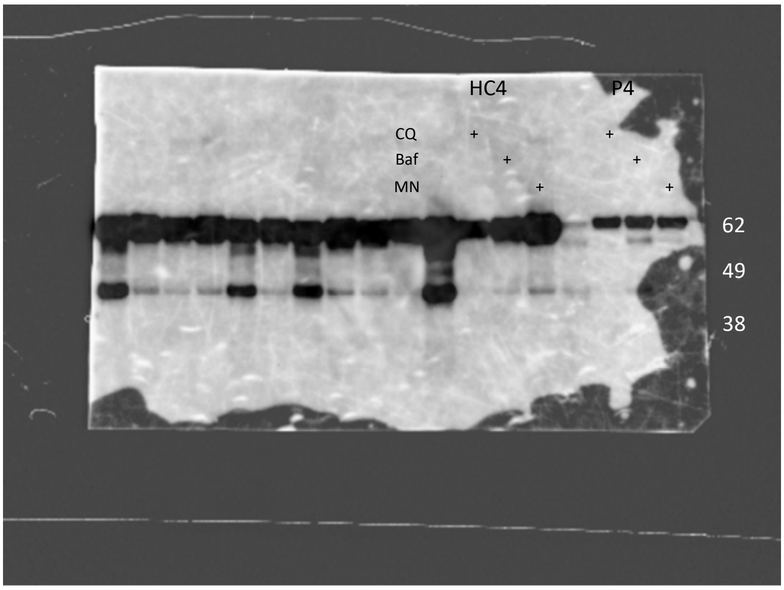

anti-β-actin (AC-15) + goat anti-mouse (#71045)

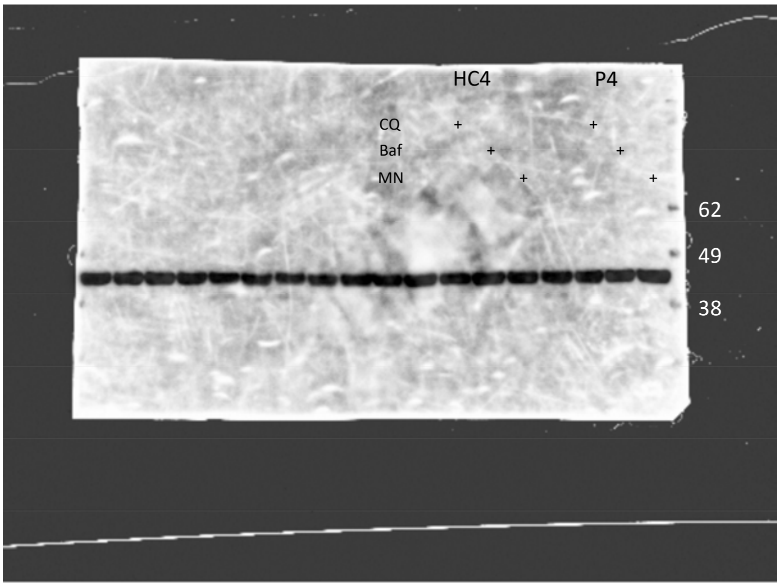

anti-ADA2 (ab288296) + goat anti-rabbit (ab205718)

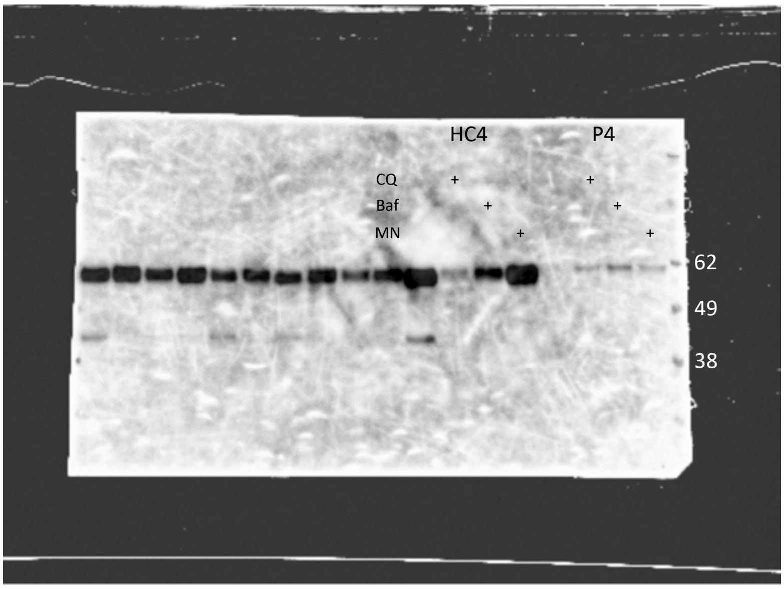

anti-ADA2 (ab288296) + goat anti-rabbit (ab205718)

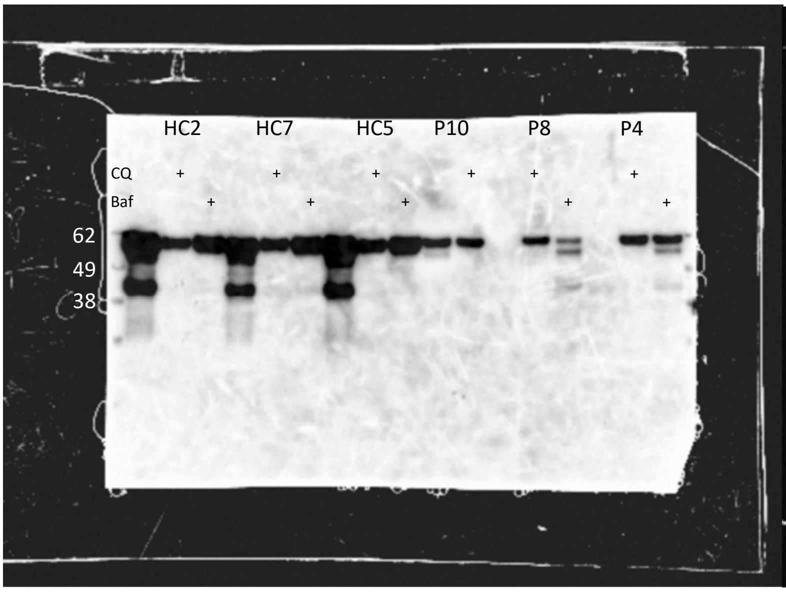

anti-β-actin (AC-15) + goat anti-mouse (#71045)

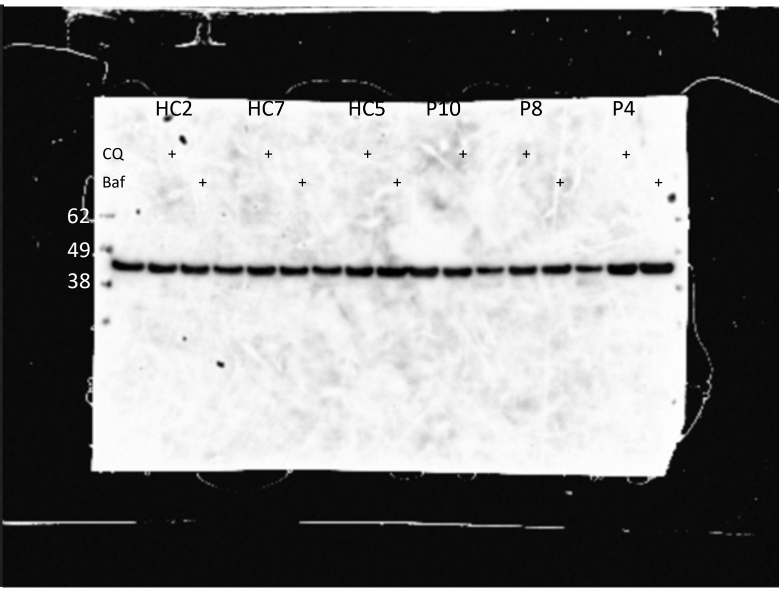

anti-ADA2 (ab288296) + goat anti-rabbit (ab205718)

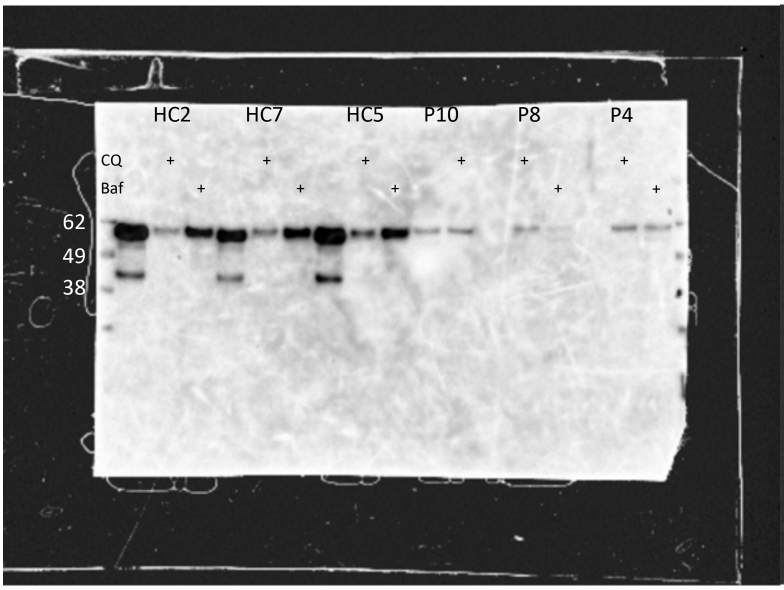

anti-LC3B [ab51520] + goat anti-rabbit (ab205718)

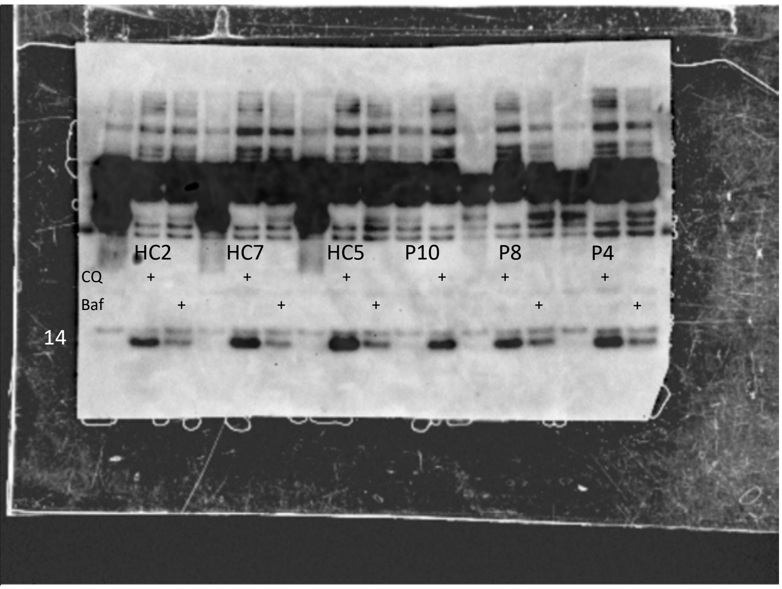

anti-ADA2 (ab288296) + goat anti-rabbit (ab205718)

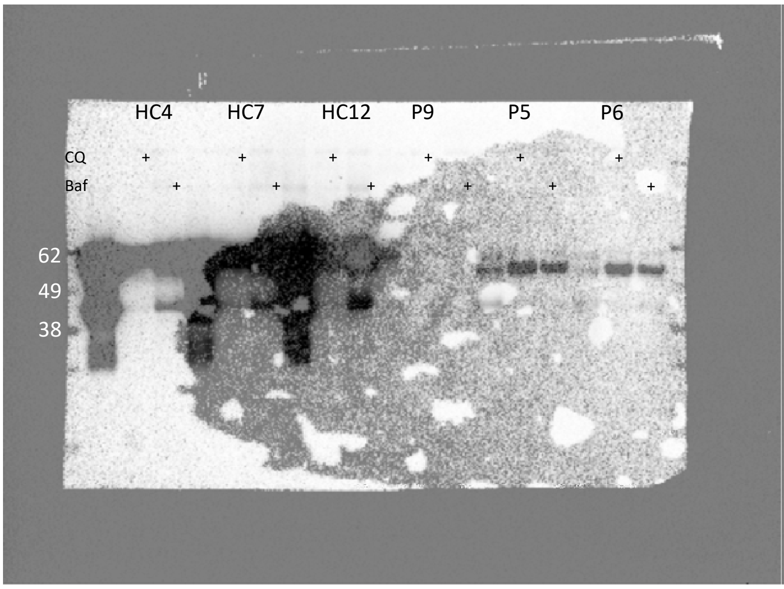

anti-β-actin (AC-15) + goat anti-mouse (#71045)

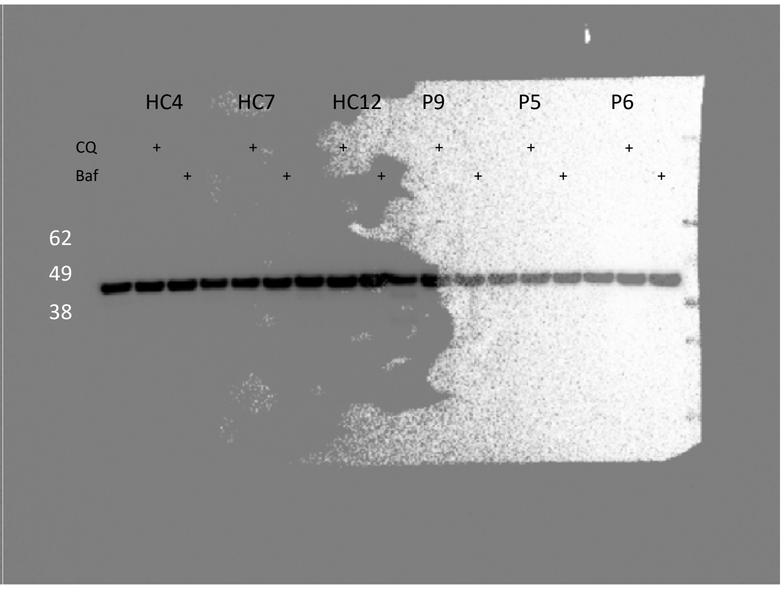

anti-ADA2 (ab288296) + goat anti-rabbit (ab205718)

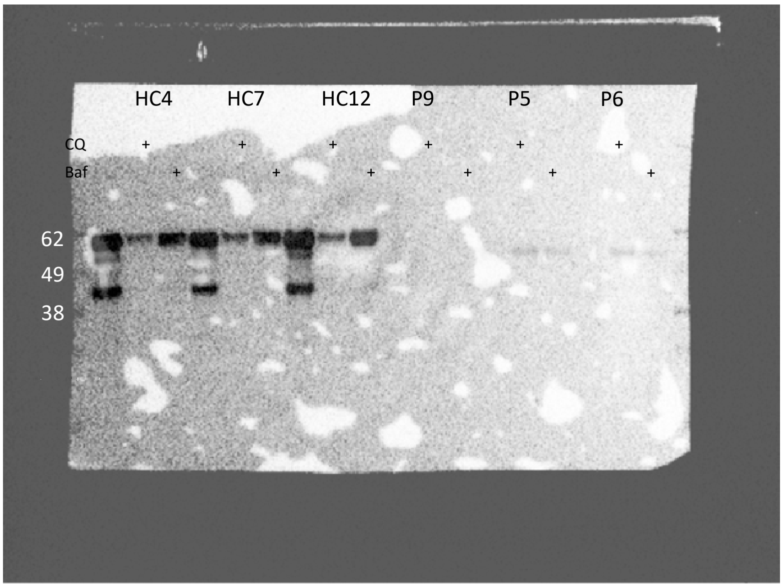

anti-LC3B [ab51520] + goat anti-rabbit (ab205718)

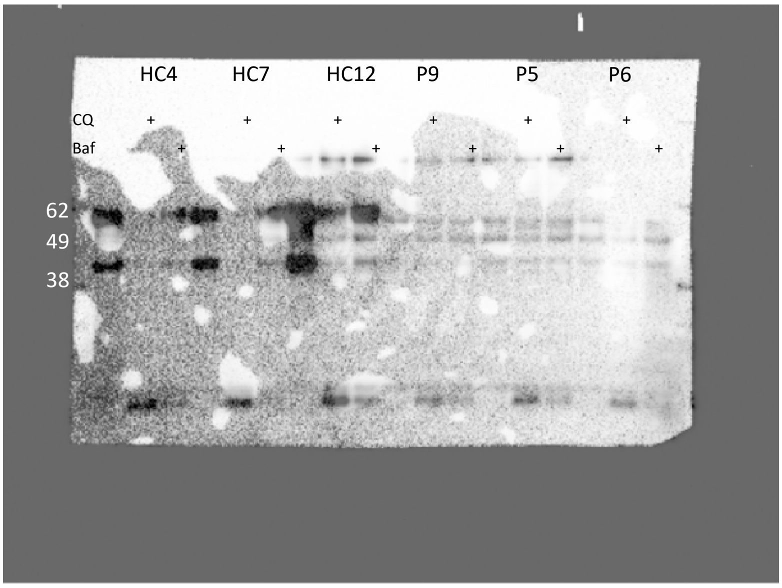

anti-ADA2 (ab288296) + goat anti-rabbit (ab205718)

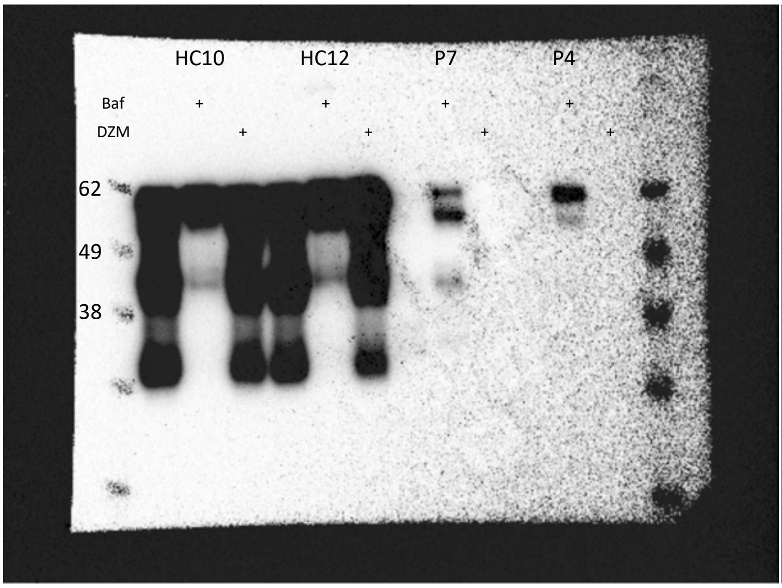

anti-β-actin (AC-15) + goat anti-mouse (#71045)

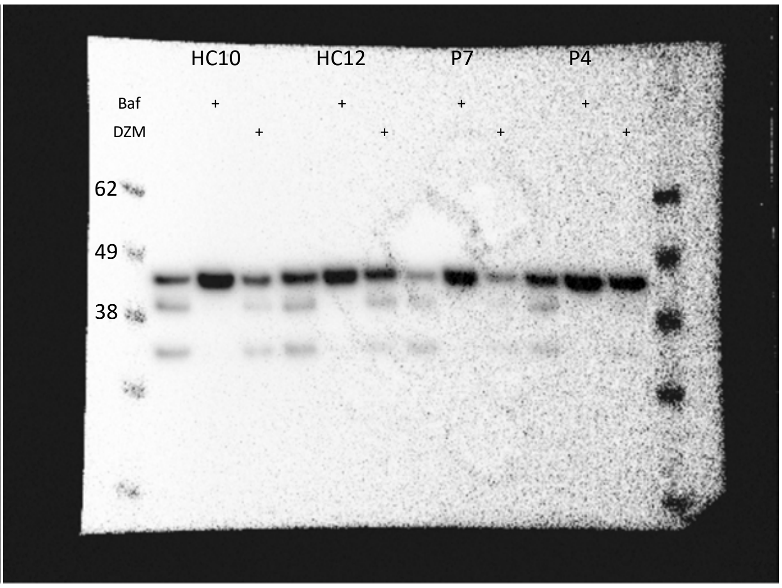

anti-ADA2 (ab288296) + goat anti-rabbit (ab205718)

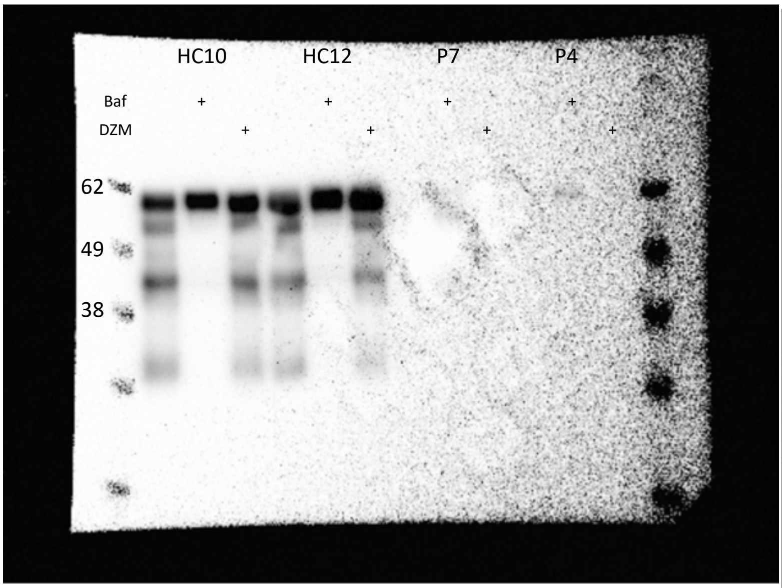

anti-LC3B [ab51520] + goat anti-rabbit (ab205718)

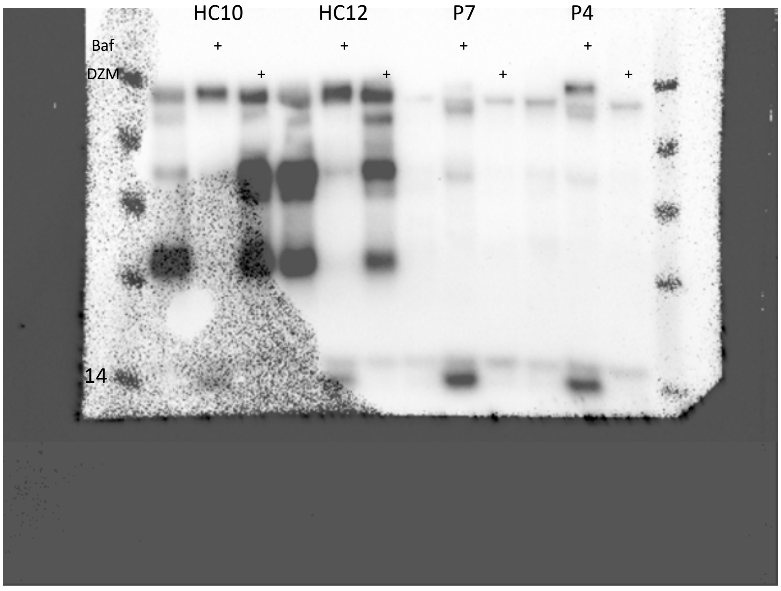

Figure 1C

anti-ADA2 (ab288296) + goat anti-rabbit (ab205718)

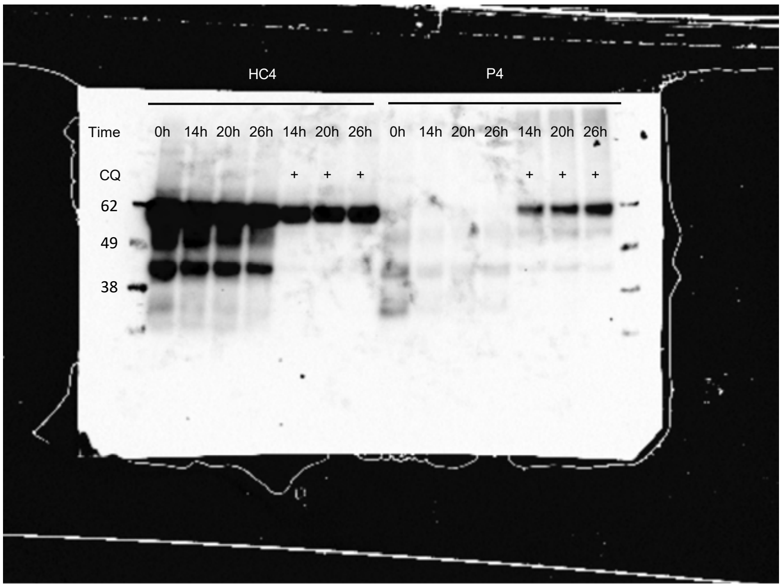

anti-β-actin (AC-15) + goat anti-mouse (#71045)

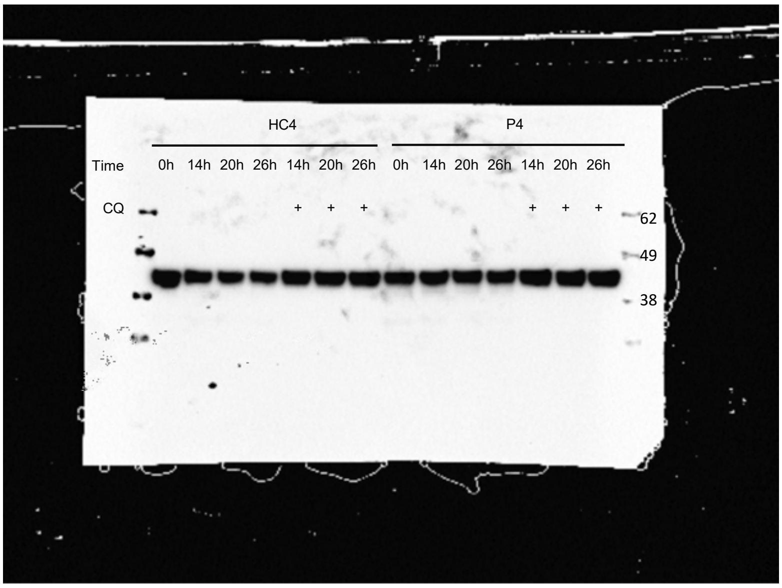

anti-ADA2 (ab288296) + goat anti-rabbit (ab205718)

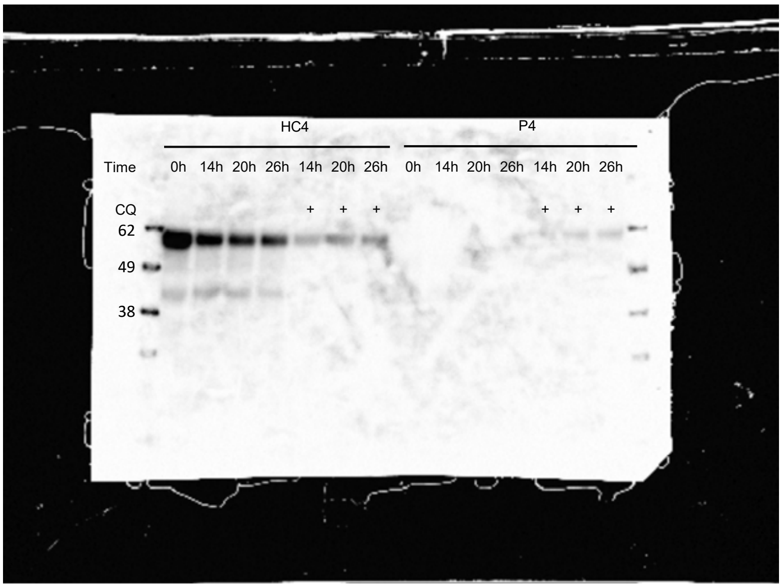

anti-LC3B [ab51520] + goat anti-rabbit (ab205718)

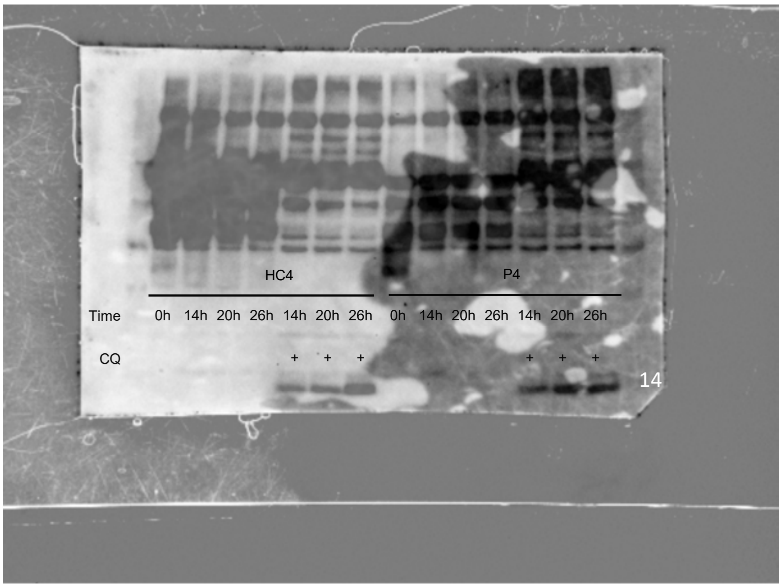

Figure 1D

WCE

anti-ADA2 (ab288296) + goat anti-rabbit (ab205718)

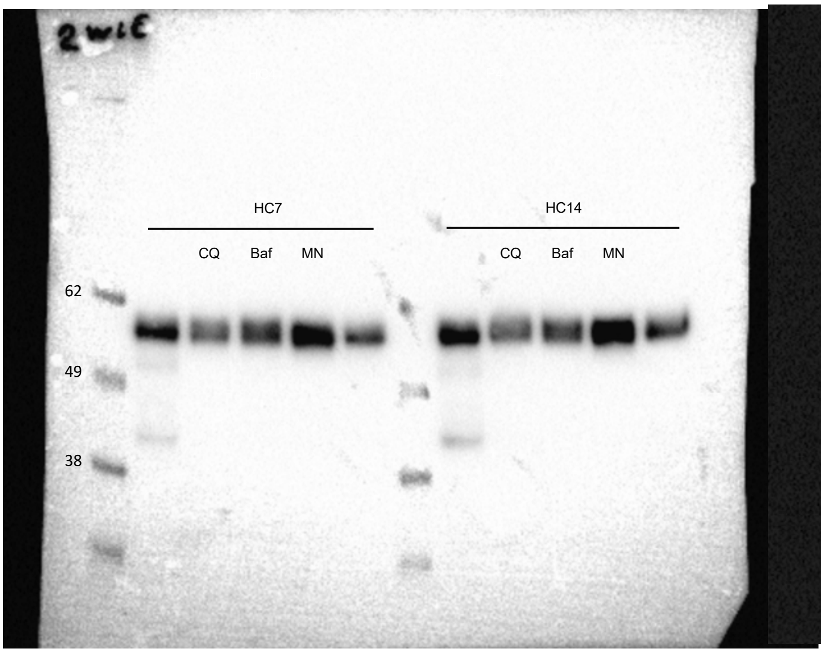

anti-β-actin (AC-15) + goat anti-mouse (#71045)

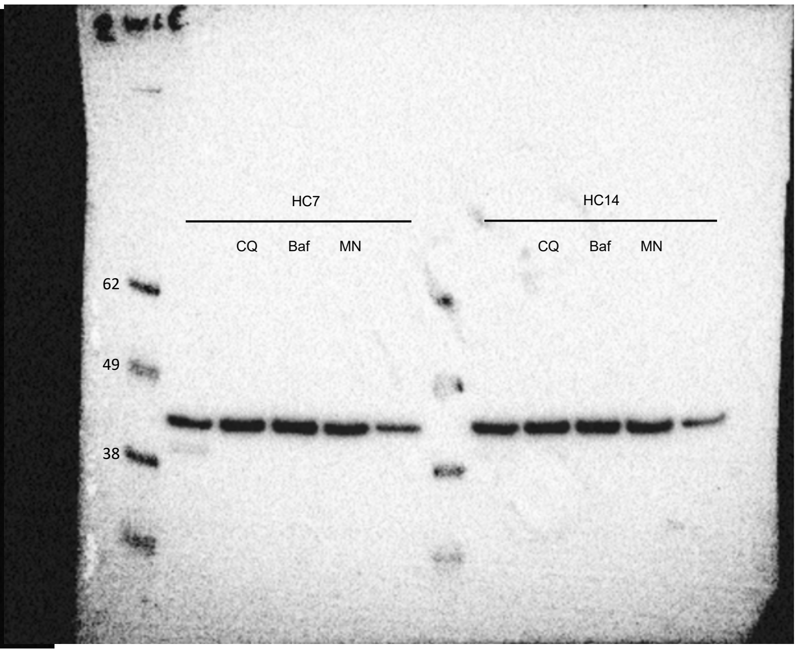

SN

anti-ADA2 (ab288296) + goat anti-rabbit (ab205718)

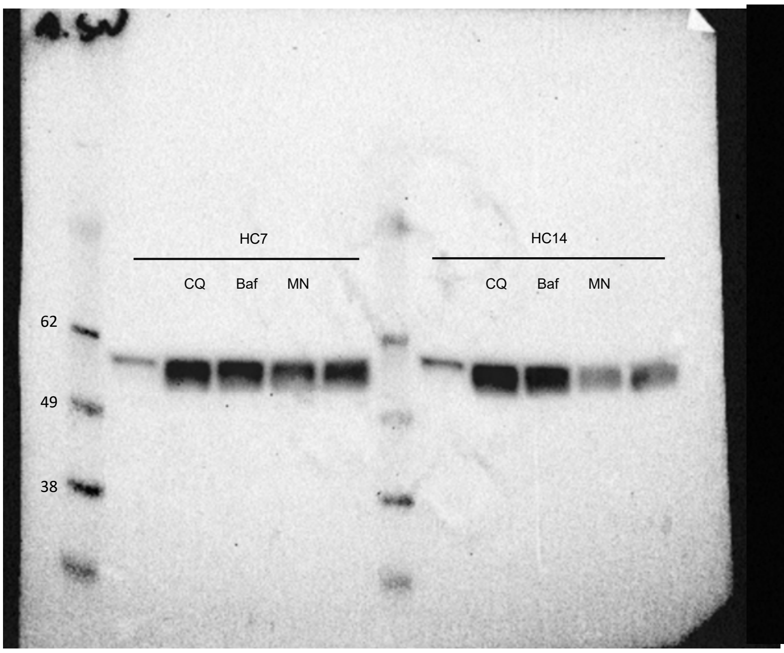

anti-β-actin (AC-15) + goat anti-mouse (#71045)

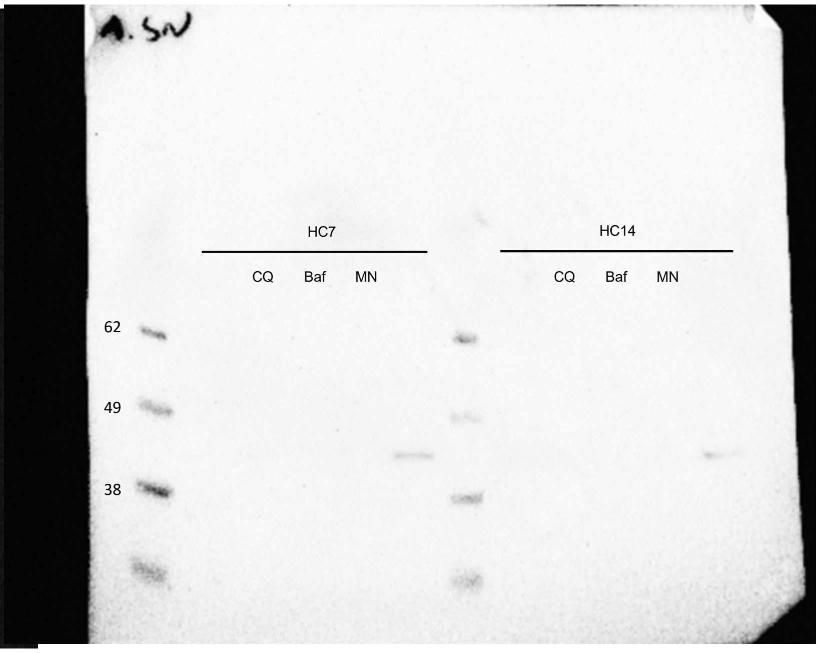

Figure 1E

anti-ADA2 (ab288296) + goat anti-rabbit (ab205718)

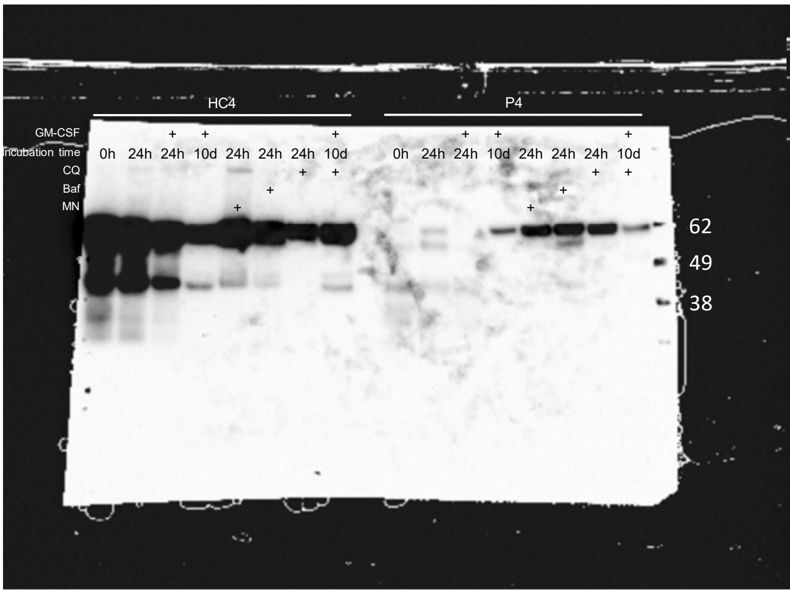

anti-ADA2 (ab288296) + goat anti-rabbit (ab205718)

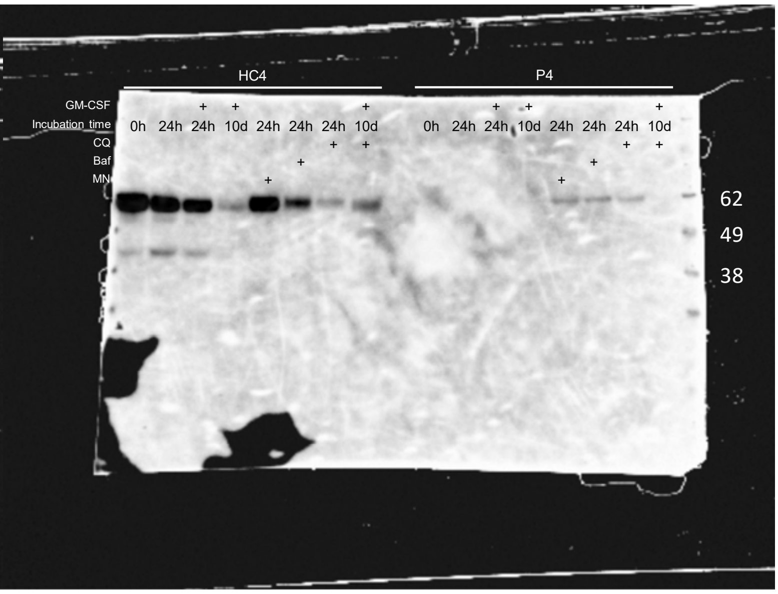

anti-LC3B [ab51520] + goat anti-rabbit (ab205718)

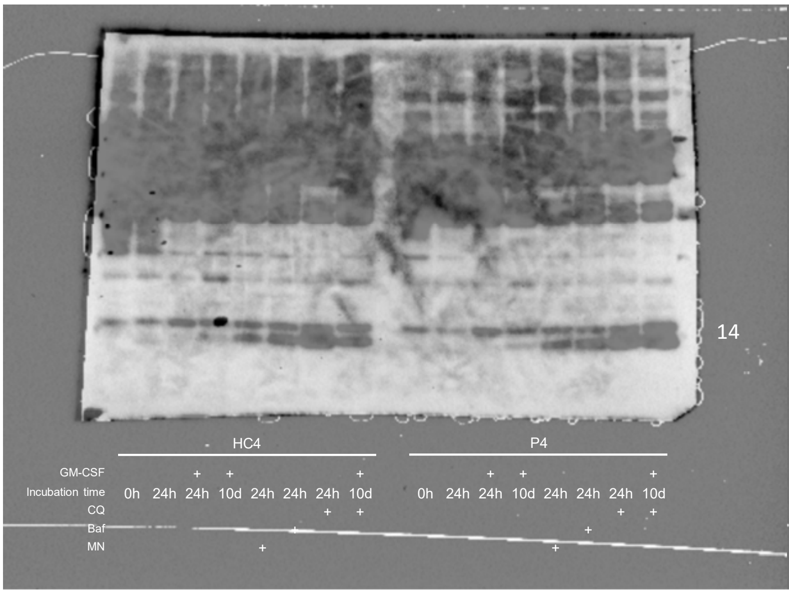

anti-LC3B [ab51520] + goat anti-rabbit (ab205718)

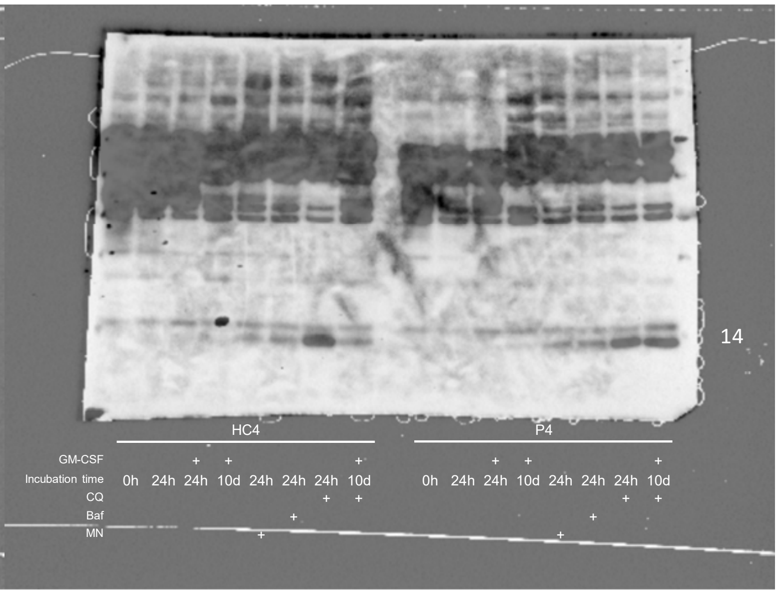

anti-β-actin (AC-15) + goat anti-mouse (#71045)

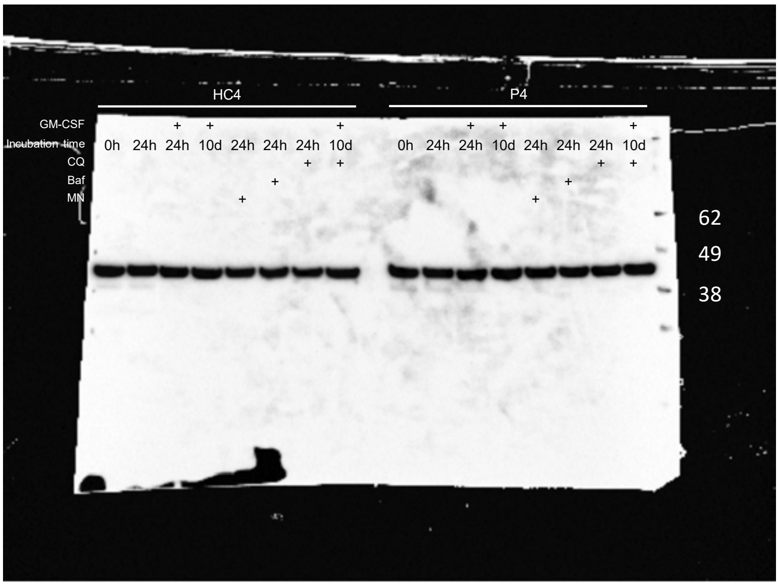

Figure 2D

anti-ADA2 (ab288296) + goat anti-rabbit (ab205718)

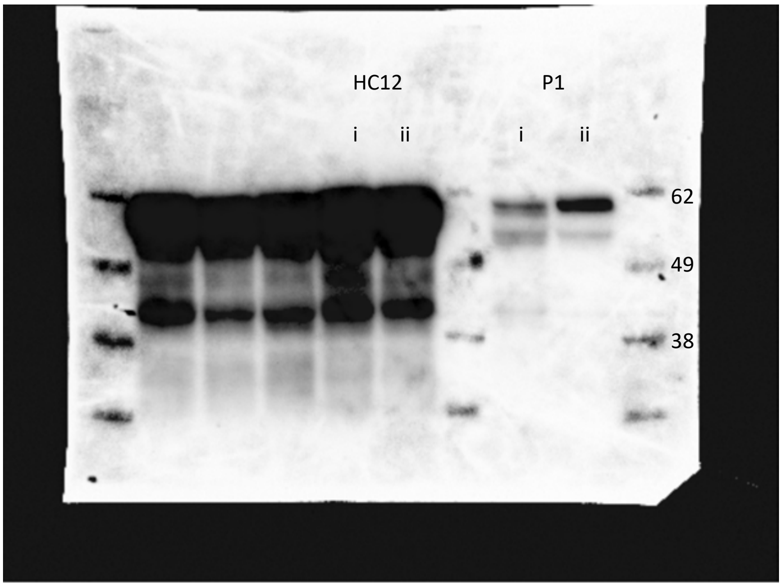

anti-β-actin (AC-15) + goat anti-mouse (#71045)

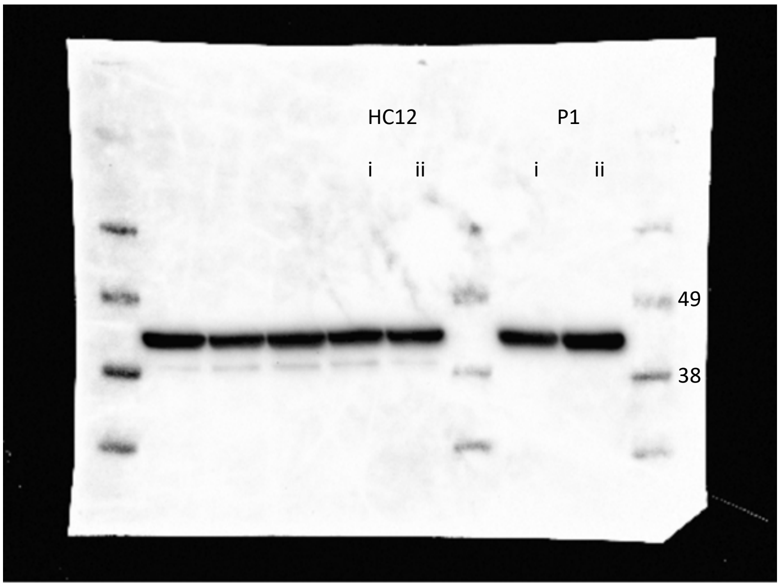

anti-ADA2 (ab288296) + goat anti-rabbit (ab205718)

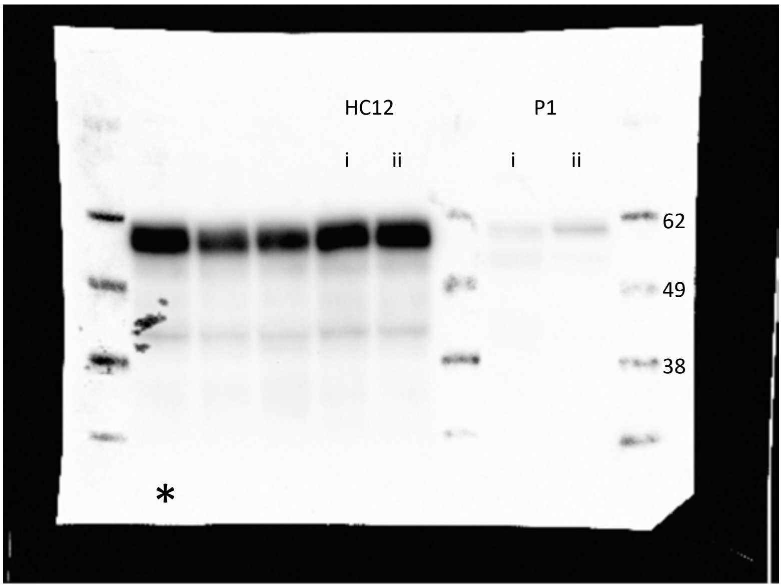

* The left part of this western blot that is not relevant to this manuscript has been published in the following article: https://doi.org/10.1101/2023.10.25.564037

Figure 3B

anti-LC3B [ab51520] + goat anti-rabbit (ab205718)

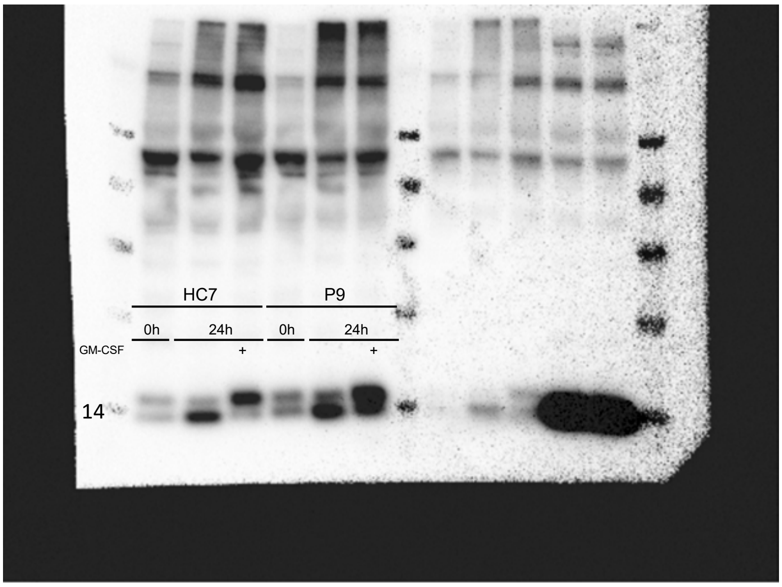

anti-β-actin (AC-15) + goat anti-mouse (#71045)

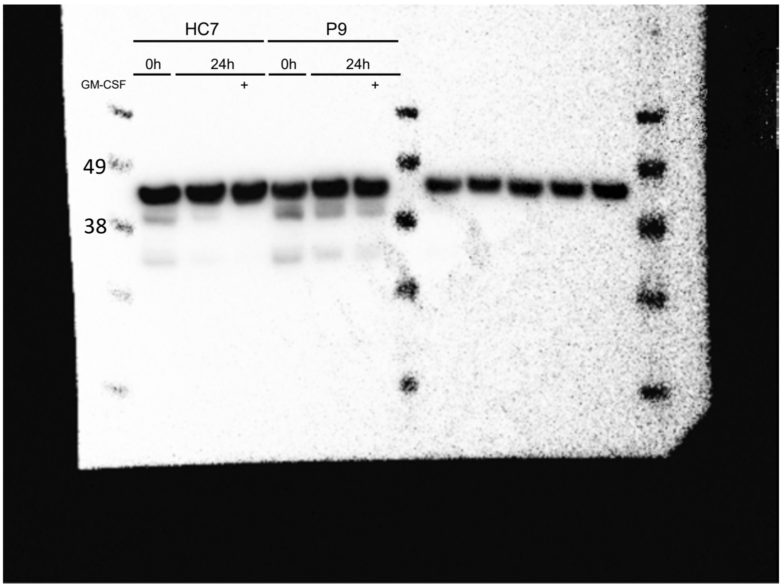

anti-LC3B [ab51520] + goat anti-rabbit (ab205718)

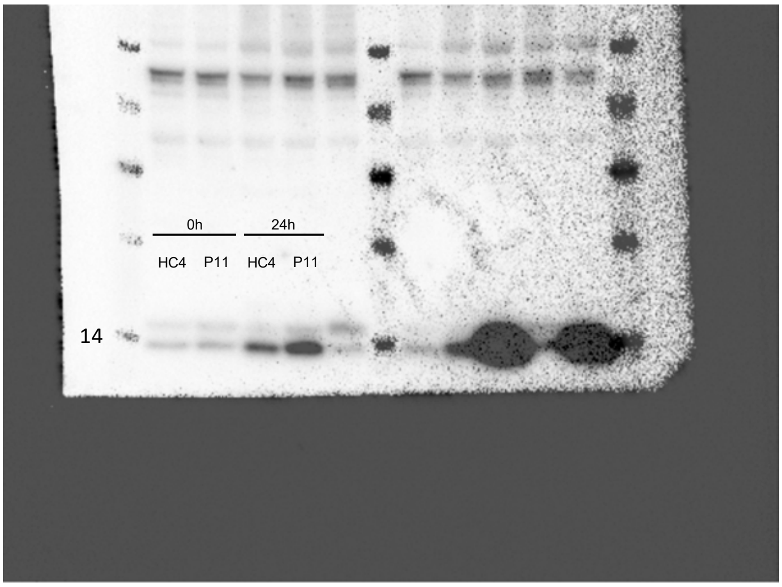

anti-β-actin (AC-15) + goat anti-mouse (#71045)

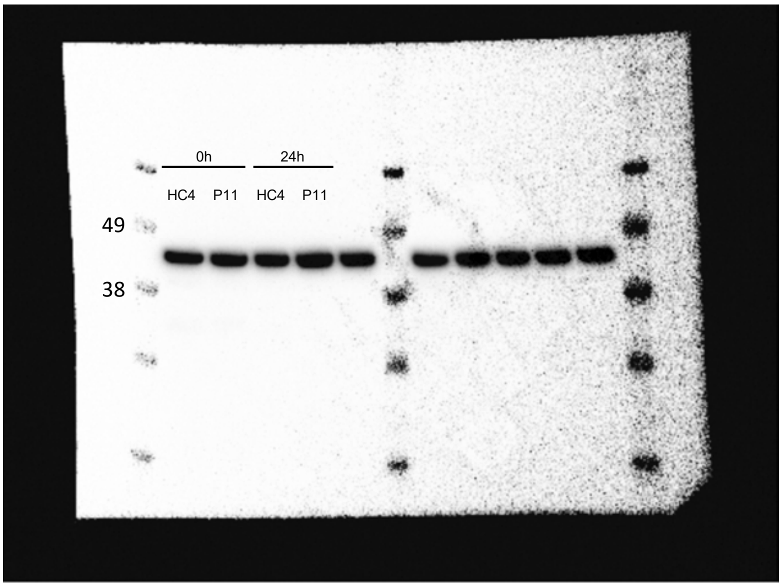

anti-LC3B [ab51520] + goat anti-rabbit (ab205718)

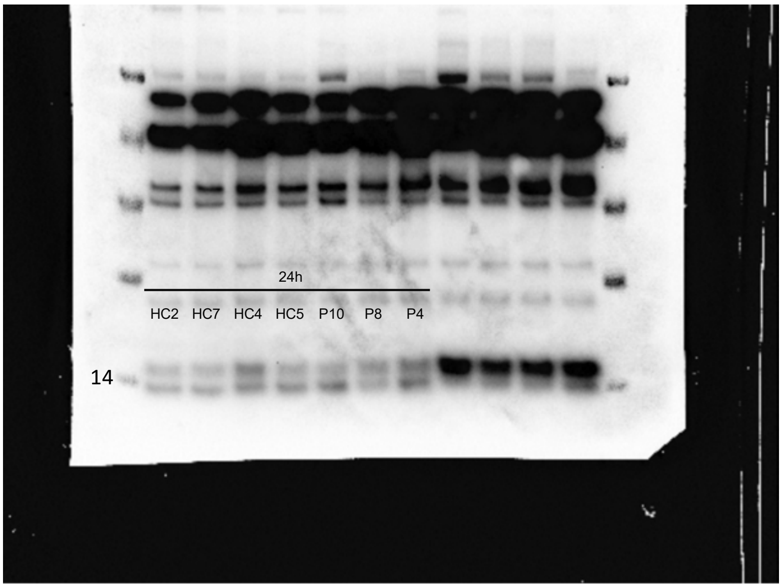

anti-β-actin (AC-15) + goat anti-mouse (#71045)

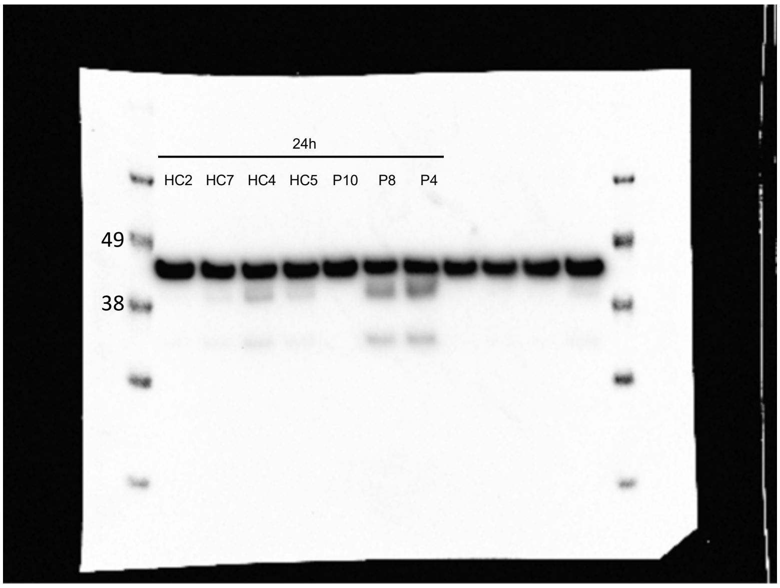

anti-LC3B [ab51520] + goat anti-rabbit (ab205718)

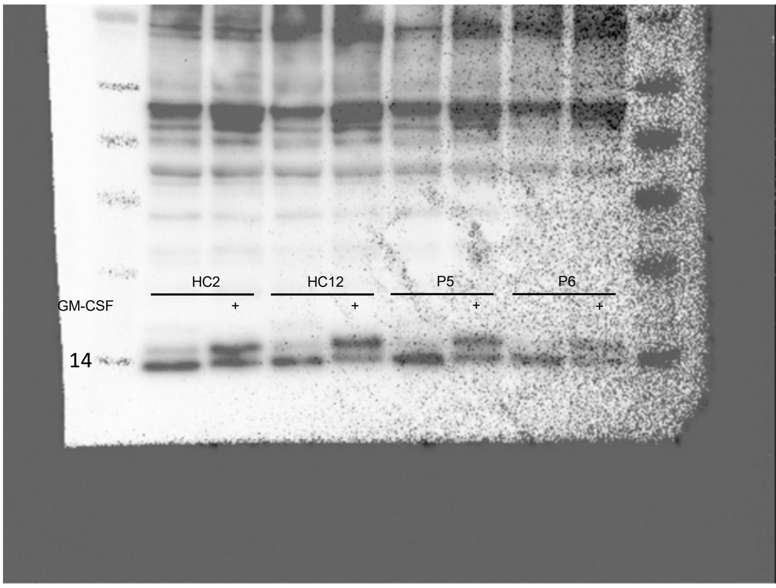

anti-β-actin (AC-15) + goat anti-mouse (#71045)

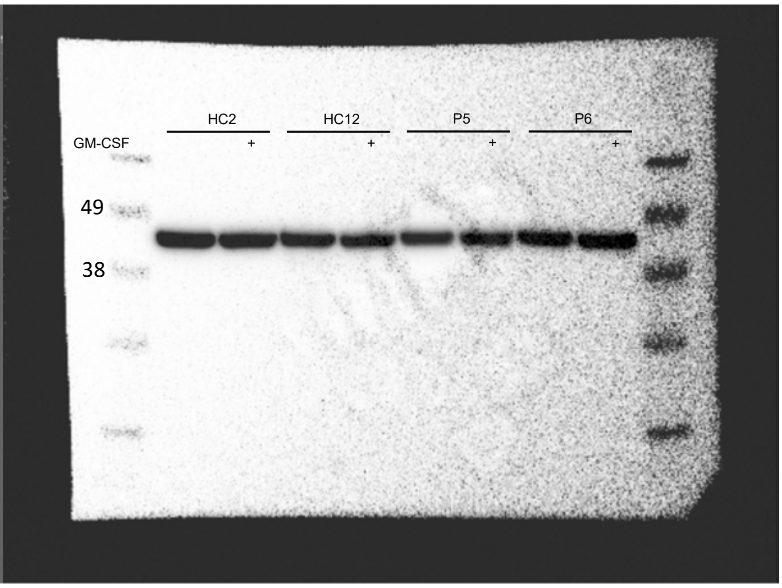

anti-LC3B [ab51520] + goat anti-rabbit (ab205718)

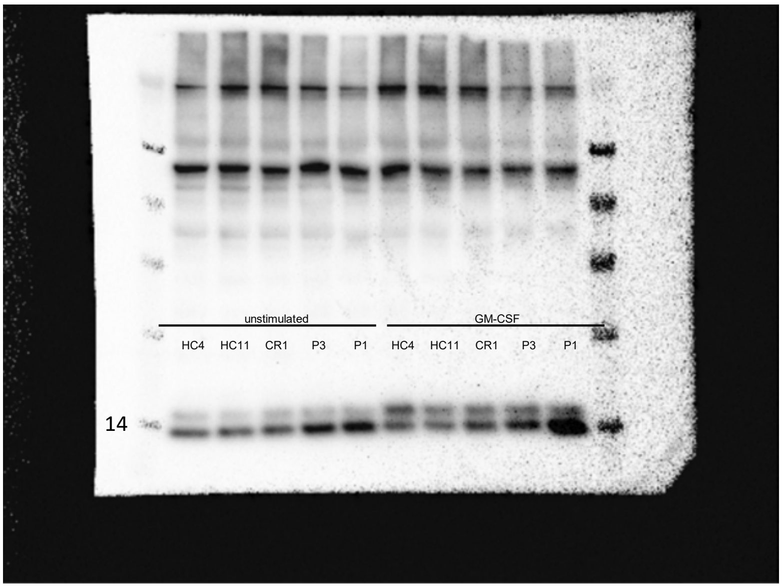

anti-β-actin (AC-15) + goat anti-mouse (#71045)

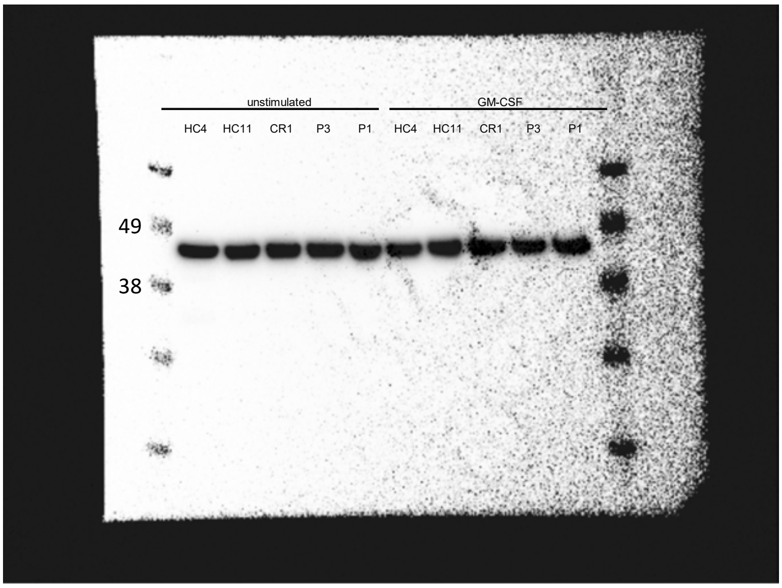

Figure 3D

Baf

anti-ADA2 (ab288296) + goat anti-rabbit (ab205718)

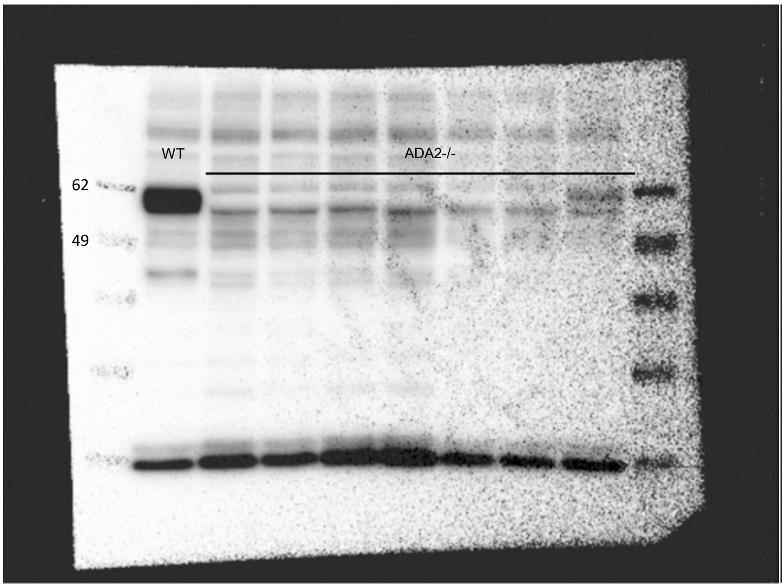

anti-β-actin (AC-15) + goat anti-mouse (#71045)

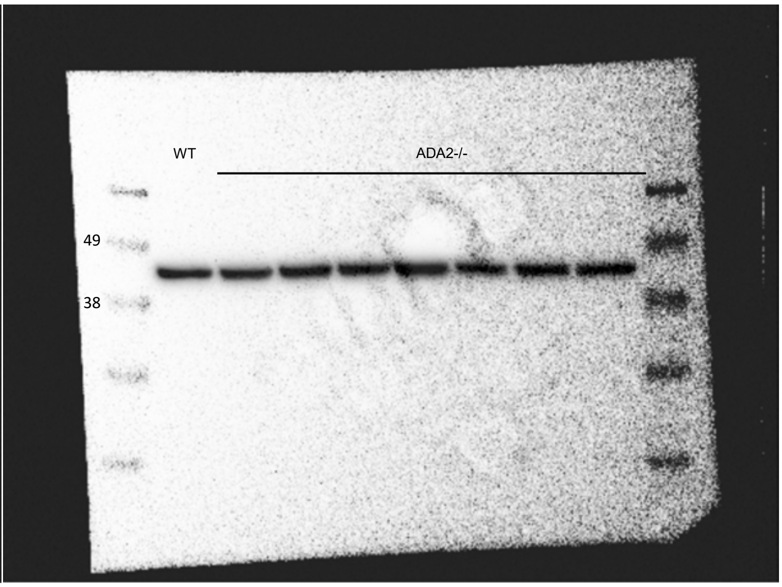

anti-LC3B [ab51520] + goat anti-rabbit (ab205718)

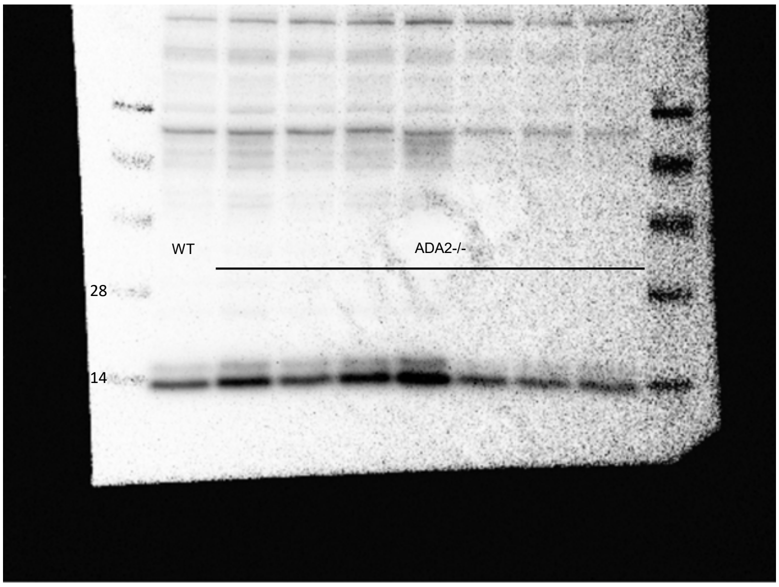

Figure 3D

untreated

anti-ADA2 (ab288296) + goat anti-rabbit (ab205718)

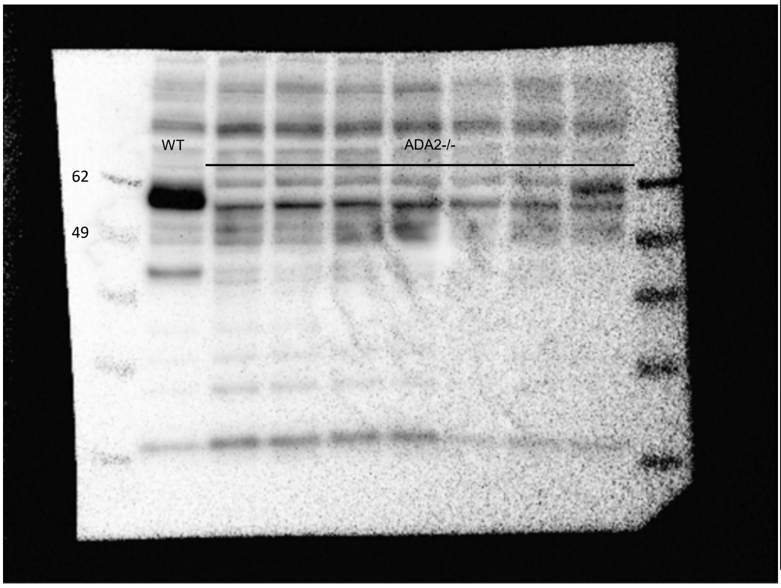

anti-β-actin (AC-15) + goat anti-mouse (#71045)

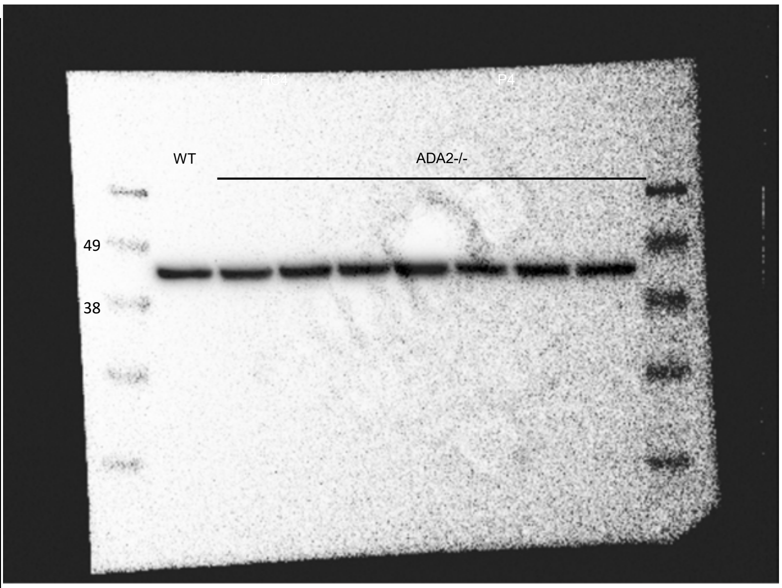

anti-LC3B [ab51520] + goat anti-rabbit (ab205718)

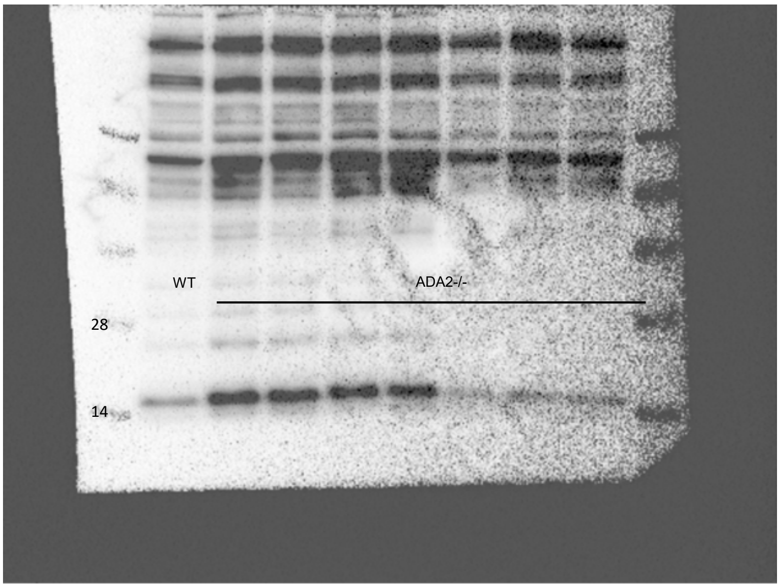

Figure 3E

untreated

anti-LC3B [ab51520] + goat anti-rabbit (ab205718)

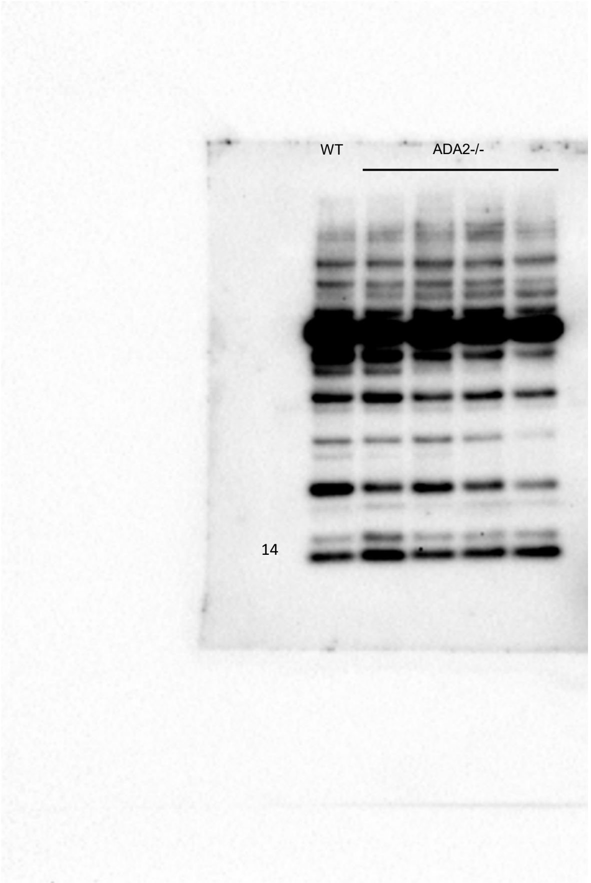

anti-β-actin (AC-15) + goat anti-mouse (#71045)

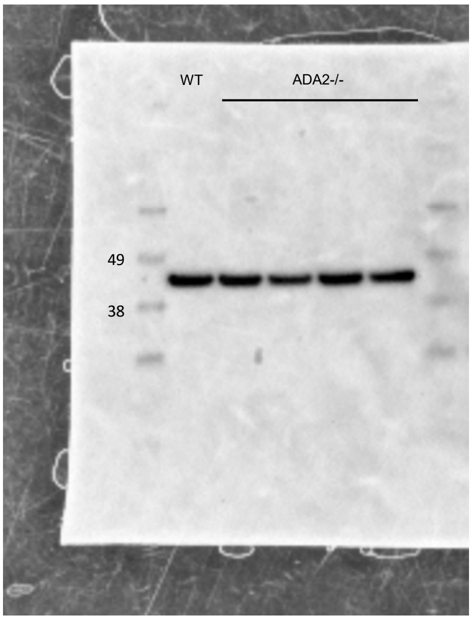

Supplement: Supplementary Figs [file mmc2.pdf]
